# Frequent Droughts Reduce Carbon Stabilisation in Organo‐Mineral Soils

**DOI:** 10.1111/gcb.70657

**Published:** 2026-01-06

**Authors:** Fabrizio Albanito, Sabine Reinsch, Mark Richards, Amanda M. Thomson, Bernard J. Cosby, Bridget A. Emmett, David A. Robinson

**Affiliations:** ^1^ UK Centre for Ecology & Hydrology Bangor UK; ^2^ Institute of Biological and Environmental Sciences University of Aberdeen Aberdeen UK

**Keywords:** biogeochemical model, carbon sequestration, carbon use efficiency, drought, heathland, organo‐mineral soil, soil carbon, soil moisture

## Abstract

Climate change is increasing drought frequency, threatening the stability of soil carbon sinks. While droughts are known to accelerate soil organic matter decomposition and enhance CO_2_ emissions, the long‐term effects of recurrent droughts on soil remain unclear. We addressed this pressing issue by modelling long‐term drought events in a temperate heathland on organo‐mineral soil using the ECOSSE biogeochemical model and developing new metrics to assess changes in soil organic carbon (SOC) sequestration and stabilisation. Across all scenarios, drought events decreased the size of microbial (BIO) and humified (HUM) SOC pools by up to 15% and 8% respectively. Short‐interval droughts weakened the BIO‐to‐HUM transfer, leading to incomplete recovery after rewetting, whereas prolonged droughts increased decomposition of stable pools at depth but allowed only partial re‐equilibration during recovery. These changes were mirrored by contrasting responses in the carbon use efficiencies of labile (CUE_I_) and stable (CUE_S_) pools. During frequent droughts, CUE_I_ remained relatively stable, while the contribution of CUE_S_ increased indicating a higher contribution of stable SOC pools under soil moisture stress. The carbon sequestration efficiency (CSE = CUE_I_/CUE_S_) declined by up to 15% under prolonged droughts compared with more frequent drought‐rewetting cycles, signalling a progressive reduction in soil carbon sequestration. The stabilisation efficiency (SE = ΔHUM/ΔBIO) declined to about 40%, implying that recurrent droughts reduced the efficiency with which microbial carbon was stabilized into the HUM pool. Collectively, these metrics revealed a reversal in the CSE‐water relationship: CSE increased with soil water during drought but declined after rewetting, indicating a persistent post‐drought decoupling between decomposition and stabilisation processes. Recurrent droughts thus reshape SOC dynamics reducing CSE and altering the balance between decomposition and stabilisation with depth. Drought frequency rather than duration, emerges as the dominant control on long‐term soil carbon stability in organo‐mineral systems.

## Graphical abstract

Climate change is increasing drought frequency, threatening the stability of soil carbon sinks. Using a biogeochemical soil model, we simulated carbon cycling in an organo‐mineral soil to understand how repeated drought and subsequent moisture recovery affect long‐term carbon storage. The results show that frequent droughts disrupt the balance between carbon decomposition and stabilisation, reducing the transfer of carbon from active to stable pools. As a result, less carbon remains locked in the soil after drought, weakening storage especially at depth. The study reveals that drought frequency ‐ rather than duration ‐ plays the dominant role in soil carbon stability, gradually reducing the ability of soils to act as carbon sinks.

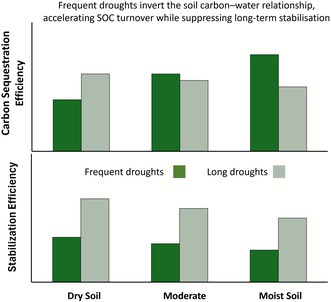

## 1 Introduction

The severity and frequency of anthropogenic drought events (Aghakouchak et al. 2021) have been increasingly linked to widespread and disruptive effects on ecosystem carbon (C) and nitrogen (N) cycling (Knapp et al. 2024; Müller and Bahn 2022). Droughts can accelerate the oxidation of soil organic carbon (SOC), increasing soil CO_2_ emissions to the atmosphere (Dong et al. 2021; Liu et al. 2022; Reinsch et al. 2017). The response of soil organic matter (SOM) to soil moisture changes is determined by mechanisms regulating its turnover at different spatial and temporal scales (Schrumpf et al. 2013). Recently, Vahedifard et al. (2024) characterized the feedback loop between climatic drought, soil desiccation, and the consequential increase in soil CO_2_ emissions. Following an initial minor increase in soil CO_2_ respiration, structural changes in the soil caused by severe drought events can increase the permeability of the soil, exposing deeper and older SOC pools to microbial decomposition (Wu et al. 2022). Over time, these changes significantly affect the diversity and functioning of microbial and macrofauna responsible for nutrient cycling and soil structure (Stovícek et al. 2017), resulting in cascading effects on ecosystem productivity, soil C sequestration, and greenhouse gas (GHG) emissions.

In this representation, resource‐limited arid and semi‐arid ecosystems are deemed to be more sensitive to droughts than more mesic ecosystems (Maurer et al. 2020). These trends are often explained by the ‘resource limitation hypothesis’ (Huxman et al. 2004; Knapp et al. 2024), which argues that productivity in wetter ecosystems is constrained mainly by nutrients or light rather than by water availability. Given that water is the primary resource limiting net primary productivity (NPP), and rates of NPP determine the amount of organic C delivered to soils through litterfall, root turnover and exudation (Tao et al. 2023), it is plausible to assume that changes in SOC sequestration depend on how quickly plant inputs decline as dry ecosystems become drier (Knapp et al. 2024; Zhou et al. 2022). However, in the literature the effect of drought on the soil C balance is more uncertain. Very wet ecosystems with C‐rich soils and anoxic soil conditions can also lose C during drought (Cleveland et al. 2010; Costa et al. 2023; Deng et al. 2021; Evans et al. 2021; Sowerby et al. 2008; Zhong et al. 2020). Changes in deep‐profile moisture can further alter SOC stability by shifting the balance between mineral‐associated organic matter (MAOM) and fresh organic inputs (e.g., root exudates), with a potential tipping point occurring where precipitation equals or exceeds evapotranspiration (Heckman et al. 2023). Such subsurface moisture–mineral interactions may therefore modulate the direction and magnitude of soil C responses to drought across contrasting ecosystems.

Understanding whether drought‐induced changes lead to soil C losses or gains requires assessing how the stability of SOC sequestration responds to several extrinsic factors, such as drought timing, severity, and intermittency that jointly influence above‐ and below‐ground biotic and abiotic properties controlling the sensitivity of SOM to changes in soil moisture (Sierra et al. 2015; Masuda et al. 2024; Robinson et al. 2019; Wunderling et al. 2022). While drought experiments and meta‐analyses have provided useful estimates of the impacts of drought on a wide range of ecological responses (Knapp et al. 2024), they often capture short‐term effects and do not quantify the cumulative or frequency‐dependent impacts of recurrent droughts on soil C dynamics. Process‐based models can help bridge this gap by integrating short‐term experimental evidence with long‐term biogeochemical feedbacks. In this study, we combine observational data from a seasonally water‐logged in situ climate change experiment (Domínguez et al. 2017; Emmett et al. 2004; Reinsch et al. 2017; Seaton et al. 2022; Sowerby et al. 2008, 2010) with simulations from the ECOSSE biogeochemical model (Smith et al. 2010a, 2010b) to explore how drought frequency affects the capacity of organo‐mineral soils to retain C. ECOSSE was used as a diagnostic framework linking field observations to soil processes that mediate the turnover and stabilisation of distinct SOM pools with different decomposition rates. By varying the recurrence interval of a six‐year observed climate cycle, we generated scenarios that isolate the influence of drought frequency while maintaining comparable hydrological conditions.

Our analyses focus on three complementary aspects of the soil C response to drought: (i) the C sequestration efficiency (CSE), defined as the relative balance between respiration from labile and stable SOC pools; (ii) depth‐resolved changes in microbial biomass (BIO) and humified organic matter (HUM); and (iii) the stabilisation efficiency (SE), defined as the proportion of BIO converted into HUM rather than released as CO_2_. Combined, these metrics allow us to test whether recurrent droughts produce reversible hydrological effects or instead generate persistent imbalances among SOC pools that weaken C stabilisation after rewetting. In addition, through this approach, we provide a mechanistic assessment of how drought frequency governs the redistribution of C within the soil system and identify model‐based early warning signals of declining CSE in organo‐mineral soils.

## 2 Methods

### 2.1 Site Description and Experimental Treatments

The experimental site used in this study is a *Calluna vulgaris* (L.) Hull dominated hydric upland Atlantic heathland, located at Clocaenog forest in NE Wales, United Kingdom. Details relating to the site were described in detail in several studies (Emmett et al. 2004; Seaton et al. 2022). Here, we summarize the main characteristics of the site, experimental treatments, and information used in the simulations of the ECOSSE model (see Text S1 in Supporting Information).

The soil type is a podzolic organo‐mineral soil classified as a Ferric stagnopodzol in the Hafren Series in the Soil Survey of England and Wales (Hallett et al. 2017). The soil profile has four horizons in total (Table 1). At the top, the organic layer of on average 7 cm of litter and fragmented plant residues (LF) is distinct from a horizon of illuvial organic matter accumulation (Oh), followed by a Mulky‐Modified Mineral horizon of approximately 10 cm which overlays a mineral Gley soil layer that reaches saprolite shale at the bottom of the soil profile at a maximum of 32 cm depth.

**TABLE 1:** Characteristics of the soil profile at the Clocaenog experimental site. The vegetation is dominated by *Calluna vulgaris* , and the soil consists of a C‐rich topsoil layer 7 cm deep and approximately 50% of soil organic carbon (SOC), and an underlying layer of gley subsoil ~25 cm deep with SOC content ranging from 11% to 3%. In the table, soil depth (cm), BD = bulk density (g cm^−3^), SOC stock (t C ha^−1^), particle‐size fractions of clay, sand and silt (%). Texture measurements of the organic LF horizon were not possible due to its high soil organic matter content.

Soil type	Soil horizon	Soil depth	SOC	BD	Clay	Silt	Sand
Organic	LF	5	31.3	0.11	0.00	0.00	0.00
Organic	Oh	7	25.7	0.16	0.75	29.05	20.10
Mulky‐Modified Mineral	E	17	15.1	0.37	4.51	55.22	40.20
Mineral (Gley)	BC	32	13.0	0.85	9.73	50.07	40.20

The experiment, established in 1998, comprises replicated (*n* = 3) 4 × 5 m plots under control and drought treatments. Drought is imposed during the growing season (March–September) by automated retractable curtains excluding 20%–26% of rainfall, while temperature manipulations (not analysed here) run in parallel. Continuous meteorological observations include precipitation (P) and air temperature (TA). Monthly potential evapotranspiration (PET) was derived using the Thornthwaite method (Thornthwaite 1948) via the SPEI R package (Vicente‐Serrano et al. 2010). Hydrological characterisation of the site, including monthly water balance (WB) analysis and seasonal classification of soil water storage, surplus, utilisation, and deficit, was performed using the Hydromad R package (Bai et al. 2009; Table S1, Figure S2).

Vegetation at the site is an old‐aged heathland community with no structural or age differences between control and drought plots. Above‐ and below‐ground biomass and litterfall (g biomass m^−2^ yr.^−1^) were collected every 6 months in each plot (Reinsch et al. 2017, 2015d; Sowerby et al. 2008). As described in Reinsch et al. (2017), total soil CO_2_ effluxes (Rs, kg CO_2_‐C ha^−1^) were measured on a fortnightly to monthly basis at the soil surface throughout the year using three permanent soil collars (10 cm diameter) placed in each plot. No aboveground vegetation was allowed to grow inside the collars. Annual soil respiration was calculated from fortnightly soil respiration measurements averaged at a monthly timescale. It is important to note that the simulations of soil CO_2_ flux from ECOSSE correspond to heterotrophic respiration (Rh, kg CO_2_‐C ha^−1^). To this end, the comparison of measured total Rs and modelled Rh required the partition of Rs into the autotrophic (Ra) and Rh fractions. Based on literature information on the temporal variability of Ra in similar heathland ecosystems (Kopittke, Tietema, et al. 2013; Kopittke, van Loon, et al. 2013), we assume that Rh accounts for 46% of Rs in summer (June–August), 52.5% in spring and autumn (March–May, September–November), and 59% in winter (December–February). To assess the robustness of this assumption, a sensitivity analysis was performed in which Rh fractions for the drought treatment were varied by ±5% and ±10% while control fractions remained fixed. This test quantified the uncertainty associated with Rs partitioning and its effect on model data comparison.

### 2.2 Modelling Drought Events

For the model simulations we selected 6 years of field observations from January 2009 to December 2014. During this experimental period, climate, soil CO_2_ fluxes, soil moisture were continuously measured in both the drought and control plots (Reinsch et al. 2015a, 2015b, 2015c). The impact of drought is simulated in four modelling scenarios with different intermittence of the drought events. In the control simulations, the 6‐year experimental period is repeated for 32 cycles, generating a simulation period of 192 years. Individual scenarios are then created, whereby in the 192 years of simulation the conditions observed in the control plots are interrupted and replaced with the conditions measured in the drought treatment. Here the drought conditions are imposed for 12, 24, 48, and 96 years. Recovery is allowed for the same length of time as the drought, meaning that if a drought is imposed for 12 years, a recovery period of12 years is simulated before a new drought period starts. Overall, this modelling approach generates four modelling scenarios (Table 2) characterized by increasing intermittence of droughts and post‐drought phases where SOC pools are allowed to recover from drought effects. A full recovery of SOC pools means reaching SOC pools observed under control conditions.

**TABLE 2:** Model scenarios used to simulate the impact of drought events on the soil system. Each scenario consisted of a total simulation length of 192 years. During the simulation, the control conditions were interrupted imposing the experimental drought conditions measured at the Clocaenog experimental site. The length of the drought and post‐drought recovery and stabilisation (PDRS) phases depends on the number of droughts imposed in the simulation.

Modelled scenario	N. of drought events	Length of drought	Length of PDRS
12‐year	8	12 years	12 years
24‐year	4	24 years	24 years
48‐year	2	48 years	48 years
96‐year	1	96 years	96 years

Depending on the specific state of the variables reported, drought‐induced changes in the SOC pools at each temporal point in the simulations were calculated as the difference between predicted values under the experimental drought conditions and the control (or baseline) conditions. The SOC analysis focused primarily on the soil C cycle and on the dynamic simulations of plant inputs (kg C ha^−1^), total SOC stock, and the size of its corresponding SOC pools (kg C ha^−1^), and CO_2_ (kg CO_2_‐C ha^−1^).

**FIGURE 1:**
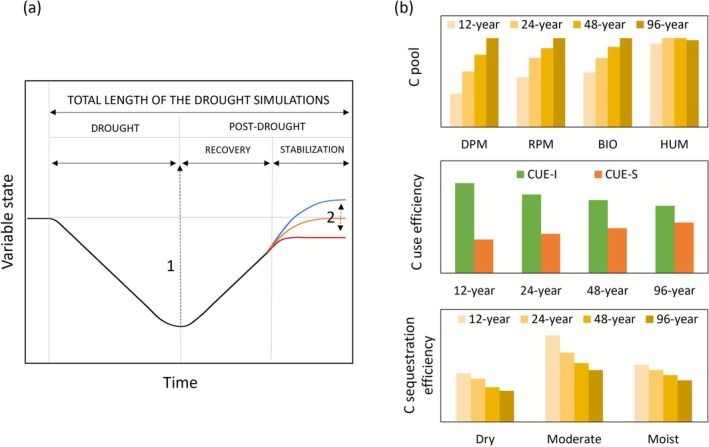
(a) Schematic of the trajectory of simulated changes induced by drought events (adapted from Müller and Bahn (2022)). During the length of the drought event (left side of the plot) the state of a variable is altered by a specific rate of change that ends when the drought event is terminated. Following the maximum impact of the drought event (dotted arrow 1), the post‐drought phase starts and continues for the same length of the drought period (Table 2). The post‐drought phase ends irrespective of whether the variable has fully recovered, changes (orange trajectory) or the impact of the drought has resulted in a shifted equilibrium trajectory (blue or red trajectories), reflecting a loss or a gain in SOC pools (dotted arrows 2) when compared to the initial baseline state estimated under control conditions. (b) Conceptual synthesis of the modelled responses of soil‐carbon pools (DPM, RPM, BIO, HUM), carbon‐use efficiency for the decomposition of fresh inputs (CUE‐I) and stable pools (CUE‐S), and carbon‐sequestration efficiency (CSE) to increasing drought frequency (12‐, 24‐, 48‐, 96‐year) and soil water conditions (Dry, Moderate, Moist).

Changes in SOC pools were analysed using the framework described in Müller and Bahn (2022), which separates the temporal impact of droughts into drought and post‐drought recovery and stabilisation (PDRS) phases. Here, the drought and PRDS phases have the same length that vary based on the simulation scenario reported in Table 2. At the end of the drought phase, we report the maximum impact of the drought on the state of specific variables (Figure 1a). The PRDS phase starts after the drought phase and ends after the drought‐induced changes level‐off and stabilize to the original or a new equilibrium state. We assume that in the long‐term simulations, ECOSSE would always return to the state estimated for control conditions (i.e., prior to the drought event). To this end, the equilibrium achieved in PDRS depends on the intermittency of the drought events, which corresponds to the sum of the length of drought and PDRS.

### 2.3 Carbon Use Efficiency and Carbon Sequestration Efficiency

The impact of drought on soil C is explored through the response of Rh to soil moisture. A detailed description of the ECOSSE model and its mechanistic approach is provided in Smith et al. (2020) and the Section Text S1 in the Supporting Information. A brief summary of the pool‐type approach used in the model to simulate the cycling of soil C and N, plant organic C inputs, GHG emissions and soil water fluxes is provided here. In the mineralization‐immobilization cycle, the OM inputs entering the soil are partitioned between decomposable plant material (DPM) and resistant plant material (RPM). These pools decompose releasing CO_2_, and forming microbial biomass (BIO) and humified organic matter (HUM). The relative size of DPM and RPM pools depends on the decomposability of plant C input, and their decomposition initiates the stabilisation of soil C into the BIO and HUM pools. Over time, additional CO_2_ is released from the progressive decomposition and transformation of BIO and HUM.

SOC sequestration in ECOSSE is evaluated by relating Rh to the changes in size of the different SOC pools. This allows us to distinguish between fast‐cycling SOC pools that represent fresh OM inputs (DPM + RPM) and more stable SOC pools (BIO + HUM). Following He et al. (2024), we operationally define C‐use efficiency (CUE) as the ratio between Rh and the total C fluxes transferred among SOC pools that are mediated by microbial processes, excluding physically driven transfers (e.g., sorption, aggregation, or leaching). Building on this framework, we separate two distinct fractions of Rh: CUE‐input and CUE‐storage. We define the index CUE‐input (CUE_I_) as the ratio of CO_2_ respiration derived from labile SOC pools (DPM + RPM) to their total C content, and CUE‐storage (CUE_S_) as the corresponding ratio for stable SOC pools (BIO + HUM). In other words, CUE_I_ represents the respiration efficiency of fresh organic‐matter inputs, whereas CUE_S_ reflects the respiration efficiency of more stabilized SOC pools. Taken together, these indices describe the relative behaviour of labile and stable C turnover and sequestration. To integrate their combined effects, we define the ratio of CUE_I_ to CUE_S_ as the C sequestration efficiency (CSE) of the soil system. CSE corresponds to an integrative metric that tracks how drought alters the balance between short‐term C turnover and long‐term C stabilisation potential in the ECOSSE model. An increase in CSE indicates that, per unit of available substrate C, CO_2_ respiration associated with labile pools (DPM + RPM) is stronger than that associated with stable pools (BIO + HUM). While a decrease in CSE indicates the reverse: a proportionally stronger contribution from the stable pools. Figure 1b illustrates the conceptual framework of the potential responses of CUE_I_, CUE_S_ and CSE to different drought scenarios and soil water conditions.

### 2.4 Drought Effects on Soil Carbon Sequestration

During repeated drought and recovery cycles, variations in soil water directly alter decomposition rates within these pools, modifying the balance of CO_2_ derived from labile (DPM + RPM) versus stable (BIO + HUM) pools. These substrate‐driven shifts are subsequently captured in the CUE indices (CUE_I_ and CUE_S_) and their ratio (CSE), allowing the ECOSSE model to diagnose how SOC pool turnover dynamics influence CUE under different drought frequencies and post‐drought recovery times (Table 1). To ensure that comparisons across drought scenarios were not confounded by differences in soil water distributions, CSE values were compared only at identical absolute soil water contents. Modelled soil water availability was grouped into 2‐mm intervals, and within each interval, the median CSE was estimated for each drought scenario. The 96‐year scenario was used as the baseline because, by the end of the simulations, it reached a quasi‐equilibrated state and provided consistent overlap in soil water distributions with the shorter drought‐intermittence scenarios.

To facilitate interpretation, soil water was divided into three zones corresponding to the process domains defined by the ECOSSE soil moisture modifier (see Figure S1). Below field capacity (< 80 mm) was classified as “Dry” conditions, as soil water potential falls within the inhibitory range of the ECOSSE moisture function. Under these conditions, microbial respiration and substrate turnover are limited by moisture, and decomposition of both labile and stable SOC pools is depressed. Values between 80 and 100 mm of soil water were classified as “Moderate,” representing a transitional zone where water availability supports optimal microbial activity and a balanced partitioning between mineralization and humification. Above 110 mm, “Moist” conditions approach or exceed field capacity; under this wetter state, the ECOSSE moisture modifier declines, representing oxygen limitation and the suppression of Rh.

The contribution of labile and stable SOC pools to soil C sequestration was further assessed by analysing depth‐specific patterns. In each modelling scenario, pool sizes at the end of the PDRS phase were expressed as percentage differences from control conditions for each 5‐cm soil layer. Persistent negative HUM deviations across the 0–30 cm profile indicate a depth‐integrated reduction in C‐stabilisation capacity rather than a temporary lag.

Finally, to assess if C from BIO is stabilized into HUM or lost as CO_2_ via Rh, we defined the changes of BIO and HUM pools in terms of the stabilisation efficiency (SE):

(1) SE=ΔHUM/ΔBIO

where Δ is the change in the HUM pool per unit change in BIO between consecutive years. Positive values indicate effective conversion of BIO turnover into a more stable HUM pool. Low or near‐zero SE values indicate that the turnover of the BIO pool is routed predominantly to CO_2_ respiration. While negative SE values outline that BIO rises while HUM falls, indicating a pool‐state imbalance (BIO‐HUM decoupling). SE was computed separately for Drought and Post‐drought phases over 5‐mm soil water intervals and across all modelling scenarios. Phases were defined by comparing absolute soil water availability in each drought scenario with the corresponding control value.

### 2.5 Statistics and Model Validation

Uncertainty in model simulations was calculated by considering the minimum and maximum values of soil and climatic input parameters assumed to have the greatest influence on the results of ECOSSE. As no estimates of standard errors or confidence intervals were available for these parameters, the maximum and minimum values were derived using general uncertainty coefficients (Gottschalk et al. 2010; Hastings et al. 2010). Table 3 summarised the average uncertainties for these parameters.

**TABLE 3:** General uncertainties estimated for the main parameters affecting SOC dynamics in ECOSSE (derived from Gottschalk et al. (2010) and Hastings et al. (2010)). The ECOSSE model was run two more times for each modelling scenario (see Table 2), using the selection of values in the table to obtain a maximum and minimum projected SOC stock (Equation (2) and (3)).

Parameter	Variable	Uncertainty	Min value	Max value
Ait temperature (°C)	Temp	±2%	Monthly Temp * 0.98	Monthly Temp * 1.02
Evapotranspiration (mm)	PET	±2%	Monthly PET * 0.98	Monthly PET * 1.02
Precipitation (mm)	P	±5%	Monthly P * 0.95	Monthly P * 1.05
Clay content (%)		±10%	Clay * 0.90	Clay * 1.10
SOC_t0_ (t C ha^−1^)		±20%	SOC_t0_ * 0.8	SOC_t0_ * 1.2

In the uncertainty analysis, we applied the following predefined arrangement of inputs:

(2) $$\mathrm{SOC}\;\max=\text{Model}\;\left({\mathrm{SOC}}_{\text{t0}}\;\max,\text{Clay}\;\max,P\;\max,\text{Temp}\;\min,\mathrm{PET}\;\min\right)$$

(3) $$\mathrm{SOC}\;\min=\text{Model}\;\left({\mathrm{SOC}}_{\text{t0}}\;\min,\text{Clay}\;\min,P\;\min,\text{Temp}\;\max,\mathrm{PET}\;\max\right)$$

where SOC_t0_ is the initial SOC stock inputted in ECOSSE, and SOC max and SOC min are the minimum and maximum values for the simulated SOC stocks, respectively.

Model uncertainty was calculated as follows:

(4) $$U\left(\%\right)=100\times \frac{\mathrm{SOC}\;\max-\mathrm{SOC}\;\min}{2\times \mathrm{SOC}}$$

where SOC, SOC min, and SOC max correspond to the lower and upper limits of the simulated SOC stocks at the end of the forward simulations as calculated in Equations (2) and (3).

We used the R package Metrica (Correndo et al. 2022) to evaluate the coincidence and association between measured and simulated values of Rh and soil water availability. In particular, we calculated the regression coefficient (*R* ^2^) to quantify correlation, the root mean square error (RMSE, Equation (5)) to quantify total error with the same units as the variables of interest, and the mean bias error (MBE, Equation (6)) to quantify systematic bias with the same units as the response variables:

(5) $$\text{RMSE}\left(\%\right)=\sqrt{\frac{1}{n}\Sigma {\left(O_{i}-P_{i}\right)}^{2}}$$

(6) $$\mathrm{MBE}=\frac{1}{n}\;\Sigma \left(O_{i}-P_{i}\right)$$

where *O*_*i*_ and *P*_*i*_ correspond to observed and predicted values, and *n* to the number of values. Negative and positive MBE values indicate overestimation and underestimation, respectively.

Differences in CSE among drought scenarios were assessed using non‐parametric tests. Within each absolute soil water zone (Dry, Moderate, Moist), we first applied a Kruskal–Wallis test to evaluate overall differences among the four drought scenarios (Table 2). When results were significant, pairwise Wilcoxon rank‐sum tests were conducted to determine which drought scenarios differed significantly from one another. P‐values were adjusted using the Benjamini–Hochberg false discovery rate (FDR) procedure to retain only statistically robust contrasts. To further identify how each drought frequency diverged from the low‐intermittence reference scenario (96‐year) under comparable hydrological conditions, paired Wilcoxon tests were performed on median CSE values aggregated within matched 10‐mm soil water bins, providing a direct comparison of CSE trends under equivalent soil water availability among scenarios. Only bins present in both compared scenarios were included in each test, and significance was determined using FDR‐adjusted *p*‐values within each soil water zone.

To determine whether the relationship between CSE and soil water availability differed between drought and post‐drought phases, we applied an analysis of covariance (ANCOVA). Linear models of CSE ~ Soil Water × Phase, where phase was either drought or post‐drought, were fitted across all drought scenarios. A significant interaction between soil water and phase denoted that the slope of CSE versus soil water availability differed between phases.

## 3 Results

### 3.1 Drought Effect During the Experimental Period

During the experimental period considered in this study, the years 2010, 2011, and early 2012 included one of the ten most significant drought events of the past 100 years in Wales, UK. This was followed by one of the wettest autumn seasons of the last 50 years in late 2012 (https://www.metoffice.gov.uk/weather/learn‐about/past‐uk‐weather‐events). Field observations showed that 2010 was the driest year, with a cumulative precipitation (P) of only 1041 mm, whereas 2012 was the wettest, with 1399 mm. This marked drought–rewetting cycle reduced the annual cumulative P by approximately 7.4% compared with the mean P during the experimental period (1278 mm) and with the long‐term climate average (1381 mm) used to initialise the ECOSSE model. Across the six‐year experimental period, the drought treatment reduced P by 23.2% in 2011 and by 43.6% in 2012, corresponding to an overall reduction of 32.6% ± 7.1%. The water balance (WB) at the site indicated that, on average, the experimental drought produced a water deficit (potential evapotranspiration, PET > precipitation, P) between February and August that was approximately four times greater than in the control (Figure S2; Table S1). Annual TA at the site was 7.1°C ± 4.3°C (mean ± standard deviation), with 2010 and 2014 being the coolest and warmest years of the experimental period (6.2°C ± 5.1°C, and 7.9°C ± 4.0°C), respectively. Compared with the control treatment, drought slightly reduced the mean TA by 2.0% ± 0.4% between 2009 and 2014.

**FIGURE 2:**
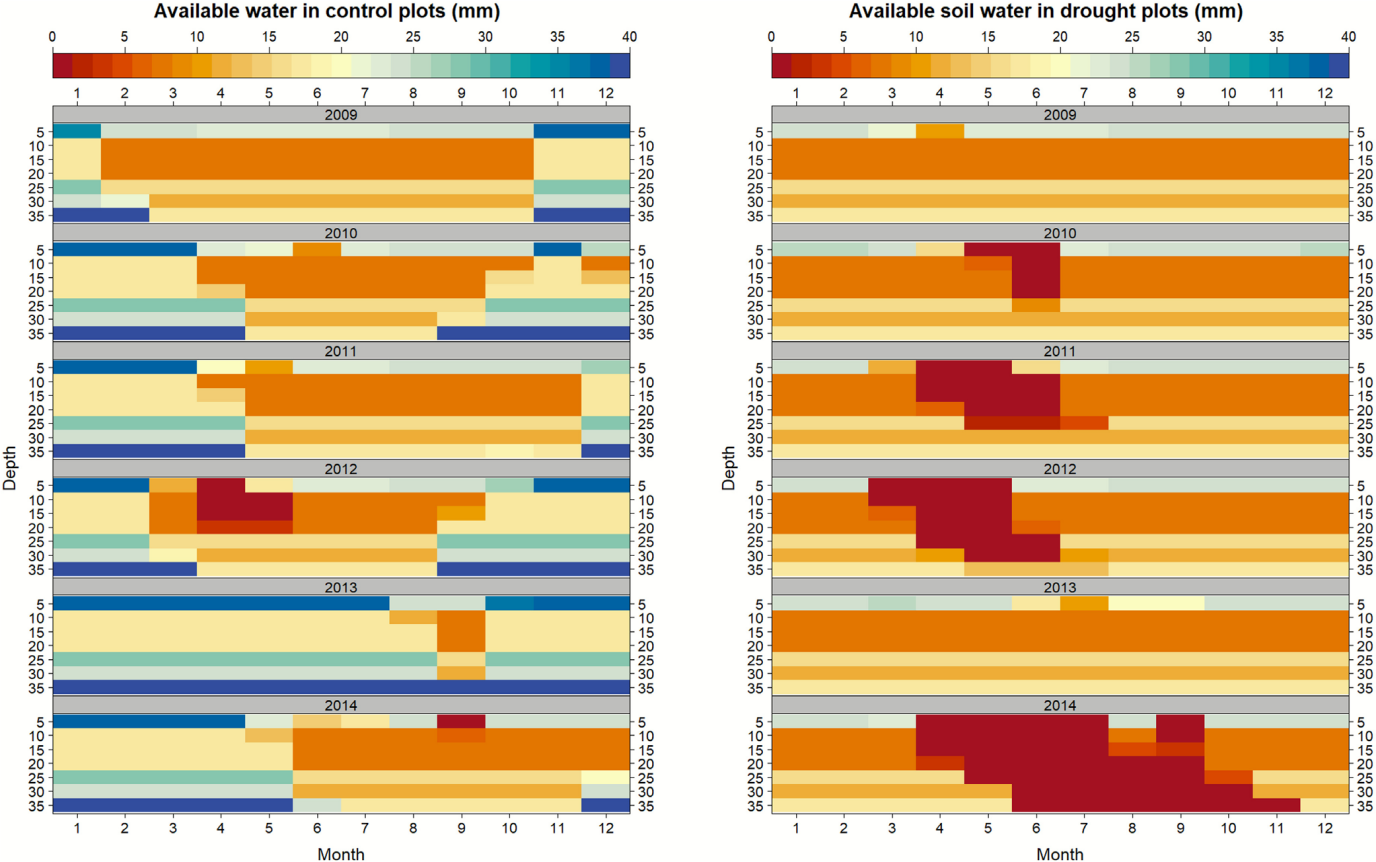
Monthly soil water availability (mm) simulated by the ECOSSE model across the soil profile every 5 cm depth intervals in the control and drought treatments corresponding to the climatic condition measured at the Clocaenog field site from 2009 to 2014.

The overall soil moisture content at field capacity estimated by ECOSSE was 75.3 mm, distributed across the soil profile as 25.0, 7.5, 7.5, 7.5, 15.6, and 12.2 mm at depths of 5, 10, 15, 20, 25, and 30 cm, respectively. During the six experimental years, soil available water in the control treatment ranged from 91.0 to 127.8 mm (mean 104.4 ± 14.1 mm), with a model uncertainty of 18.5%. In the drought treatment, soil water ranged from 60.5 to 81.8 mm (mean 62.0 ± 10.6 mm) with an uncertainty of 11.4%. Figure 2 shows the average annual soil water availability across the soil profile and the effect of the seasonal water deficit between the drought and control treatments. On average, the drought treatment reduced soil available water by 22% in 2009 and up to 60% in 2014 compared with the control.

The biomass production of the heathland vegetation community was not significantly affected by drought (Sowerby et al. 2008). Between control and drought plots, the above‐ and below‐ground living biomass, as well as the litter lying on the soil surface, increased by 7% in the drought treatment (Table S2). Field observations showed that, on average, the drought treatment increased total soil respiration (Rs) by approximately 33% ± 51% relative to the control. After partitioning Rs into autotrophic (Ra) and heterotrophic (Rh) components, monthly Rh measured in the control plots ranged from 83.5 to 470.6 kg CO_2_–C ha^−1^ (281.0 ± 114.9), and from 125.2 to 810.2 kg CO_2_–C ha^−1^ (375.1 ± 174.0) in the drought plots.

Figure S3 summarises the performance of the ECOSSE model in simulating Rh. In the control (baseline) scenario with Rh/Rs set to 0.46 in summer, 0.525 in spring/autumn, and 0.59 in winter, modelled Rh ranged from 12.3 to 1044.6 kg CO_2_‐C ha^−1^ month^−1^, with an uncertainty range of approximately 33%. Under drought conditions, Rh ranged from 49.6 to 933.0 kg CO_2_‐C ha^−1^, with an uncertainty of 24%. On average, ECOSSE overestimated monthly Rh by 56.5 kg CO_2_–C ha^−1^ in the control treatment and underestimated Rh by 20.8 kg CO_2_–C ha^−1^ in the drought treatment. Over the six experimental years, soil C simulations showed that total SOC decreased by 1458.2 ± 354.2 5 kg C ha^−1^ in the drought treatment, reducing the size of RPM, DPM, BIO and HUM by 88%, 5%, 3% and 5%. The changes applied in the sensitivity analysis, Rh/Rs fractions corresponded to proportional change of approximately ±11%, ±9%, and ±8% for summer, spring/autumn, and winter respectively (and roughly double these values for ±10%). In particular, a ±5% change in Rh/Rs caused Rh to vary by approximately ±40 kg CO_2_‐C ha^−1^ month^−1^; while a ±10% change resulted in variation of ±80 kg CO_2_‐C ha^−1^ month^−1^. Overall, these changes did not alter the direction or relative magnitude of drought–control differences. Goodness‐of‐fit metrics remained unchanged: R^2^ varied by less than 0.02 across all scenarios, RMSE differed by less than 0.05 kg CO_2_–C ha^−1^ month^−1^, and MBE remained within ±0.02 kg CO_2_–C ha^−1^ month^−1^ (Table S3).

### 3.2 Long Term Drought Effects on Soil Carbon

Following the analytical approach shown in Figure 1, the simulation results were summarised to report drought impacts on SOC as follows: (a) the maximum change or impact caused by drought, (b) the annual rate of change occurring during each drought period (i.e., 12‐, 24‐, 48‐, or 96‐year length, Table 2), and (c) the final value achieved at the end of the post‐drought recovery and stabilisation (PDRS) phase. Drought destabilised the SOC pools, with annual changes in soil C ranging from −62.8 ± 1249 to +20.6 ± 1239.6 kg C ha^−1^ yr^**−**1^ compared with the control (Table 4). Maximum SOC losses ranged from −5490 kg C ha^−1^ in the 24‐year scenario to −6231.1 kg C ha^−1^ in the 96‐year scenario, with an uncertainty of 21.5%. These SOC losses corresponded to overall changes in CO_2_ emissions from Rh, ranging from −13,425 kg CO_2_‐C ha^−1^ in the 96‐year scenario to +12,254 kg CO_2_‐C ha^−1^ in the 12‐year scenario. Therefore, an increase in drought frequency led to higher Rh, consistent with the larger annual SOC losses observed in the DPM, RPM, and BIO pools in the 12‐year drought scenario. In contrast, annual reductions in the HUM pool were greater in the prolonged droughts (i.e., 96‐year scenario). When considering the maximum drought impact across SOC pools, the 96‐year scenario caused the highest C losses in RPM, while the 12‐year scenario caused the largest loss in HUM. This distinction between the two major SOC pools highlights that the effects of soil water anomalies on SOM decomposition varied with depth within the soil profile (Table S4).

**TABLE 4:** Impact of drought phases and model scenarios on total soil organic carbon and nitrogen (SOC and SON) partitioned between different SOC pools (decomposable plant material (DPM‐C), resistant plant material (RPM‐C), soil biomass (BIO‐C), humic organic matter (HUM‐C)). SOC (mean ± SD) corresponds to the difference between drought and control treatment, with negative or positive values corresponding to a decrease or increase from control. The column “Model scenario” reports the number of years that drought is imposed in the simulations (see Table 2 for further information). Maximum impact corresponds to the highest impact of drought achieved at the end of the drought phase. Annual rates of change are separately reported for the drought and post‐drought recovery and stabilisation phase, considering the total number of years of these two phases.

Model scenario	Drought phase					Post‐drought phase				
	DPM‐C	RPM‐C	BIO‐C	HUM‐C	SOC	DPM‐C	RPM‐C	BIO‐C	HUM‐C	SOC
Control (kg C ha^−1^)	265.8 (±76.6)	31653.5 (±616.4)	1667.8 (±14.8)	59815.2 (±219.4)	100588.8 (±680.0)	263.5 (±74.9)	31528.8 (±0.0)	1661.9 (±13.2)	60305.0 (±95.7)	100945.7 (±663.5)
	Annual rate of change (kg C ha^−1^ yr.^−1^)					Annual post‐drought recovery and stabilisation rate (kg C ha^−1^ yr.^−1^)				
96‐year	19.0 (±140.1)	34.5 (±1094.5)	−1.9 (±17.6)	−31.2 (±18.4)	20.6 (±1239.6)	0.3 (±31.3)	35.7 (±220.8)	1.9 (±6.0)	16.6 (±16.5)	54.6 (±238.5)
48‐year	19.6 (±139.8)	−2.8 (±1099.7)	−3.9 (±17.7)	−28.7 (±20.3)	−15.9 (±1245.0)	0.4 (±25.5)	82.6 (±159.2)	4.3 (±5.0)	6.5 (±17.8)	93.8 (±167.9)
24‐year	20.5 (±139.0)	−55.6 (±1103.8)	−6.1 (±17.3)	−21.7 (±21.5)	−62.8 (±1249.0)	0.7 (±31.9)	136.3 (±177.4)	6.1 (±5.2)	−7.0 (±17.1)	136.1 (±197.8)
12‐year	22.6 (±137.2)	−52.3 (±1100.7)	−4.9 (±17.4)	−19.1 (±20.0)	−53.6 (±1245.8)	1.0 (±43.1)	127.0 (±244.0)	3.9 (±6.5)	−16.8 (±13.2)	115.1 (±274.3)
	Maximum impact (kg C ha^−1^)					Uncertainty (%)				
96‐year	−51.4 (±72.7)	−3140.7 (±563.5)	−170.3 (±13.0)	−2868.7 (±44.2)	−6231.1 (±646.3)	22.4	21.8	22.5	21.1	21.5
48‐year	−51.4 (±72.7)	−3121.0 (±565.5)	−167.4 (±13.0)	−2390.2 (±49.5)	−5730.1 (±652.2)	22.4	21.8	22.5	21.1	21.5
24‐year	−51.4 (±72.7)	−2847.7 (±595.8)	−146.8 (±14.2)	−2444.1 (±43.3)	−5490.0 (±684.0)	22.4	21.8	22.5	21.1	21.5
12‐year	−51.4 (±72.7)	−2469.3 (±643.5)	−126.1 (±15.7)	−3055.6 (±29.0)	−5702.4 (±725.7)	22.4	21.8	22.5	21.1	21.5

**FIGURE 3:**
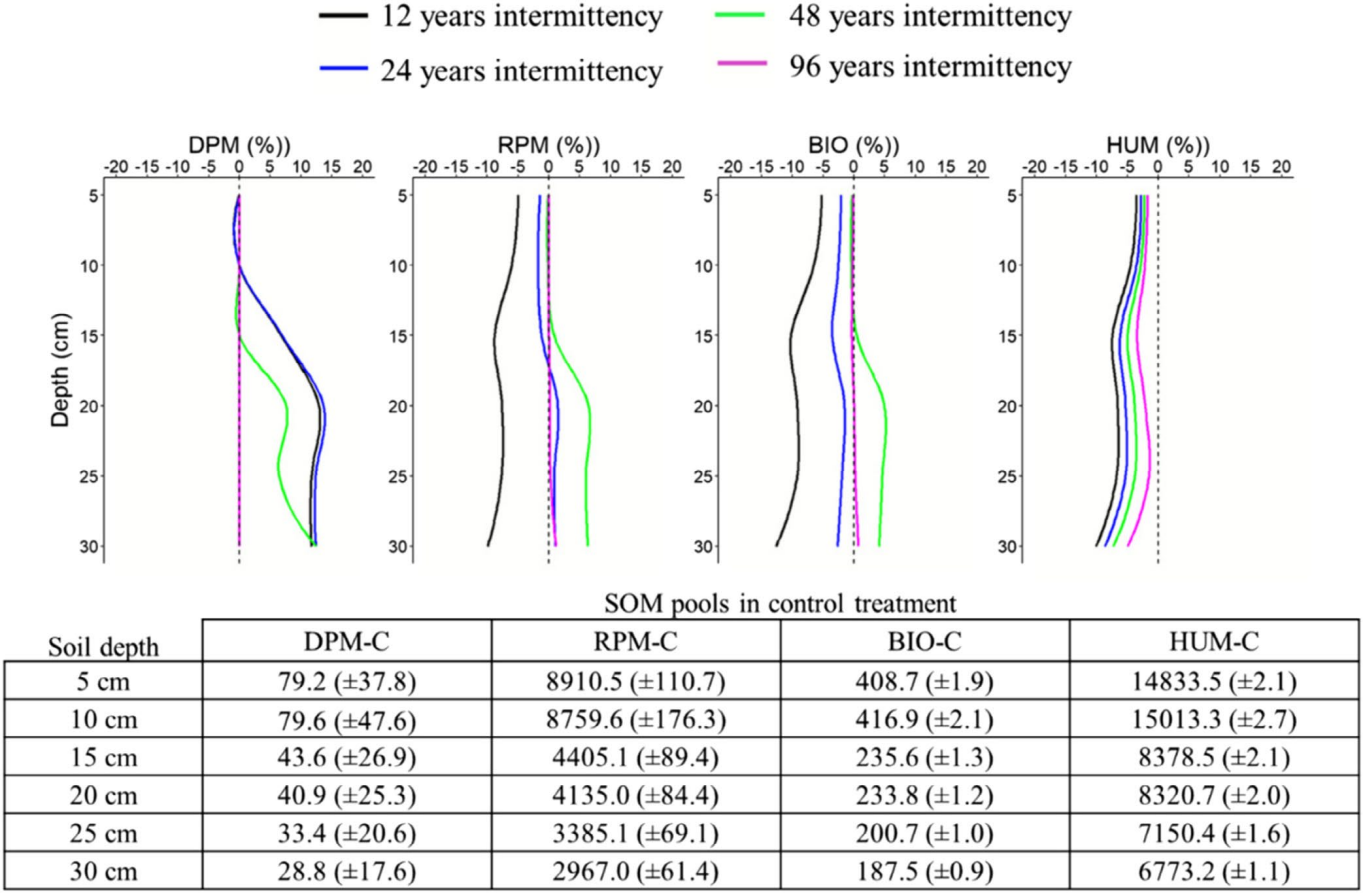
Modelled impact of drought across the soil profile on decomposable plant material (DPM), resistant plant material (RPM), soil biomass (BIO), and humic organic matter (HUM). The values in the graphs correspond to the percentage of the change at the end of post‐drought recovery (PDRS) phase in each modelling scenario, which correspond to the difference between predicted values under the drought and control conditions. A negative change corresponds to a negative impact of different drought scenarios on the final size of the four SOC pools. The size of each SOC pool simulated in the control treatment, reported in the table below the graphs, can be used to convert the percentage values shown in the graphs to the total amount of SOC change (kg C ha^**−**1^ (mean ± SD)) achieved at the end of post‐drought recovery and stabilisation phase.

Figure 3 shows the impact of soil available water changes at the end of the post‐drought recovery and stabilisation (PDRS) phase on the size of the four SOC pools. During the PDRS phase, drought conditions were interrupted to allow the soil system to recover. Across all drought scenarios, HUM was the only pool that did not recover its SOC losses, whereas DPM was the only pool that fully recovered or, in some cases, increased in size by up to 12% compared with control conditions. RPM and BIO fully recovered their C losses only in the 96‐year scenario (Figures S4–S7). In the 12‐ and 24‐year scenarios, BIO and HUM ended the PDRS phase with losses ranging from 2% to 12% relative to the control. In the 48‐year scenario, only RPM and BIO restored their initial state below 15 cm depth, where soil water anomalies were less severe.

The pattern of soil available water changes varied with the length and intermittence of the droughts. The frequency distribution of simulated values exhibited a bimodal pattern, with peaks at approximately 70 and 100 mm of soil water, indicating generally drier conditions (Figure S8b). In the 96‐, 48‐, 24‐, and 12‐year drought scenarios, soil water anomalies persisted for 101, 107, 117, and 137 years, respectively. Considering these timeframes, the 96‐year scenario showed an overall reduction in soil water of −46.6 mm (−76%) compared with the control (Table S4). Conversely, in the more intermittent drought scenarios, soil water anomalies were limited to approximately −25% compared with the control and tended to increase with soil depth.

### 3.3 Drought Effects on Soil Carbon Sequestration

**FIGURE 4:**
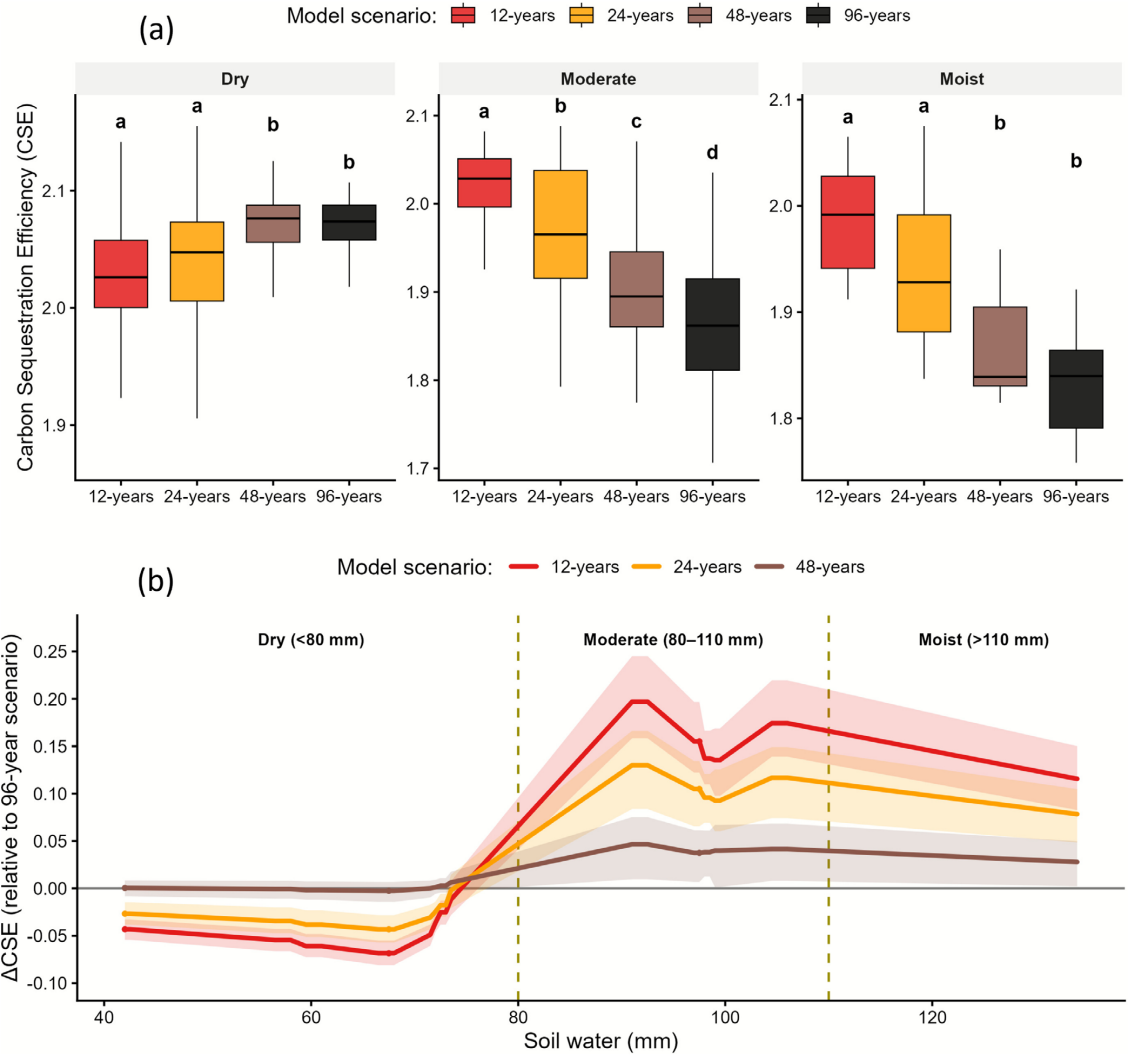
(a) Carbon sequestration efficiency (CSE) across drought scenarios within three soil water zones (Dry < 80 mm, Moderate 80–110 mm, Moist > 110 mm (Figure S1)). Paired tests compare scenarios across the same absolute 10‐mm water intervals. Letters denote groups that are not significantly different within each zone (same letter = no difference) at α = 0.05 (Kruskal–Wallis followed by pairwise Wilcoxon tests). (b) Change in CSE (ΔCSE) relative to the drought scenarios of 96 years across absolute soil water bins (2 mm intervals). Shaded ribbons show 95% bootstrap confidence intervals; vertical dashed lines indicate soil water zones boundaries (Dry, Moderate, Moist).

The relationship between decomposition of SOC pools and soil available water was analysed by examining the temporal trends of CUE corresponding to the decomposition of fresh organic‐matter inputs and SOC pools (CUE_I_ and CUE_S_, respectively). Across the full length of the simulations, both with and without soil water anomalies, we found that CUE_I_ was nearly twice as high as CUE_S_ (Figure S8a), indicating that the decomposition and redistribution of fresh inputs represented the dominant process driving soil C turnover. We then aggregated responses into distinct soil water zones to examine the patterns of CSE across different drought scenarios (Figure 4a). Under low soil water conditions, the 12‐ and 24‐year drought scenarios exhibited slightly lower median CSE than the 48‐ and 96‐year scenarios, consistent with reduced microbial‐associated (BIO) recycling under acute dryness. At moderate water availability, CSE increased progressively with shorter drought intermittence (12 > 24 > 48 > 96 years), with median differences of up to 0.15 between the extreme scenarios. At high soil water availability, short‐interval droughts again produced significantly higher CSE than prolonged droughts (letters a–b in Figure 4a; Table S5). Together, these contrasting trends outlines that increasing drought frequency sustained higher CSE during rewetting and PDRS phases, whereas prolonged droughts weakened overall CSE. However, it is important to note that although several pairwise contrasts showed significant differences in Figure 4a, none remained statistically significant after Benjamini–Hochberg correction for multiple comparisons (Table S5B). Therefore, these results should be interpreted as indicative trends rather than strict statistical separations.

Moreover, the results revealed a change in the relationship between CSE and soil water regimes (Figure 4b). Under dry conditions, changes in CSE (ΔCSE) were clustered around zero, with minor decreases (−0.04 to −0.06) for the 12‐ and 24‐year droughts relative to the 96‐year baseline. At moderate soil water, ΔCSE increased sharply to +0.18 to +0.20 for the 12‐year scenario, +0.10 to +0.15 for the 24‐year scenario, and +0.04 to +0.08 for the 48‐year scenario. Under moist conditions, ΔCSE remained positive but declined in amplitude, indicating that short and more frequent droughts still promoted higher CSE. These results highlighted a reversal in CSE sensitivity: as soil water increased above the optimal range, prolonged droughts no longer enhanced CSE after rewetting, whereas frequent drought–rewetting cycles maintained positive ΔCSE values, indicating higher sequestration efficiency relative to the baseline (96‐year drought scenario).

**FIGURE 5:**
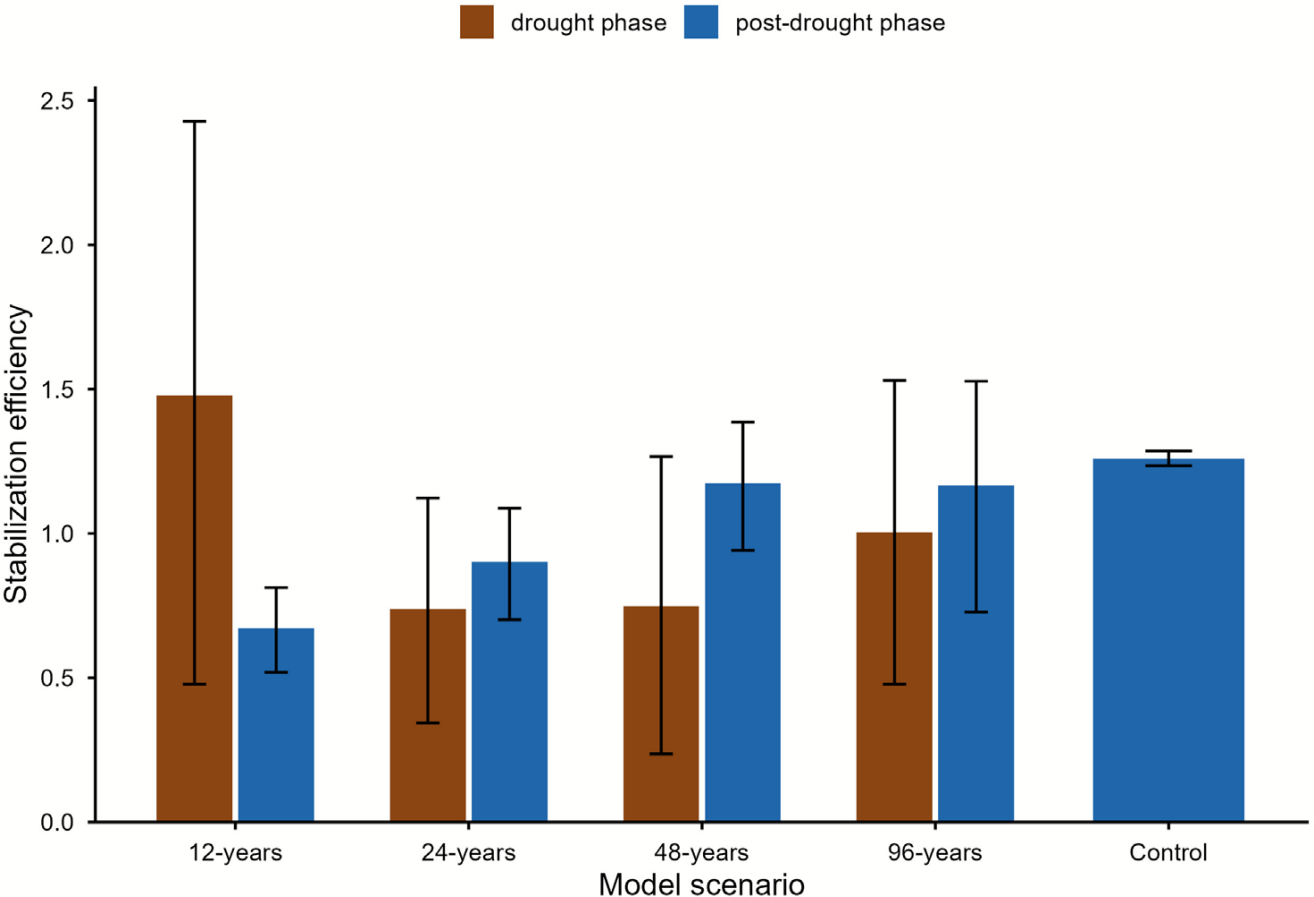
Stabilisation efficiency (SE) modelled drought scenarios and drought phases. Bars show mean SE (ΔHUM / ΔBIO) for each drought scenario (12‐, 24‐, 48‐, and 96‐years) and Control. Error bars show 95% confidence intervals from 6‐year block bootstrapping. Positive values indicate effective conversion of the BIO pool into the more stable HUM pool, while low or near‐zero SE values indicate that BIO turnover is routed predominantly to CO_2_ respiration rather than to C stabilisation in HUM.

Vertical patterns of SOC pools (Figure 3; Table S4) outlined how these differences emerged mechanistically in the ECOSSE model. The HUM pool declined under all drought scenarios, with the strongest reductions (−6% to −8%) occurring under short drought intervals (12–24 years) between 10 and 25 cm depth, where long‐term C stabilisation dominates. Intermediate and low drought intermittence (48 and 96 years) produced progressively smaller losses (~−3% to −2%), indicating partial recovery when rewetting phases were longer. The BIO pool exhibited a parallel but more variable change. Frequent droughts (12‐years) reduced the size of BIO uniformly across the profile (−10% to −15%), compared with only −2% to −5% under longer drought intervals. The concurrent declines in BIO and HUM pools confirmed that repeated drought‐rewetting cycles weakened the BIO‐to‐HUM conversion pathway, thereby limiting C stabilisation even when water availability returned to control conditions. Conversely, longer drought cycles promoted decomposition of older, deeper C pools, redistributing C from stable pools to CO_2_ respiration losses. These results were further supported by the analysis of covariance, which revealed that the soil water sensitivity of CSE differed by phase (between drought and post‐drought periods; Table S6). During drought phases, CSE increased slightly with soil water, whereas in post‐drought phases it became negative and decreased with soil water. This phase interaction was highly significant (*p* = < 0.001), confirming a phase‐dependent reversal in the CSE–moisture relationship. The analysis of stabilisation efficiency (SE) complemented this finding. Across all drought scenarios, SE remained positive (~0.6–1.5), and declined systematically with increasing drought frequency, showing the lowest values under the 12–24‐year cycles. In particular, SE declined further during post‐drought phases compared with drought periods, outlining a consistent loss of SE after rewetting in the post‐drought phase (Figure 5). This pattern suggests that although the decomposition processes resumed following moisture recovery, the conversion of BIO into HUM became progressively less effective as drought events occurred more frequently.

## 4 Discussion

### 4.1 Impact of Drought on Carbon Sequestration Efficiency

In the last decades, understanding how recurrent droughts shape soil C cycling has become a central research focus. Across ecosystems, the soil scientific community largely agrees that under prolonged and extreme drought conditions changes in the interactions between plants, soil organisms, and abiotic factors (Reinsch et al. 2024) can lead to cascading effects that ultimately affect soil C sequestration (Cardozo et al. 2024; Cordero et al. 2023; Müller and Bahn 2022; Vahedifard et al. 2024). However, the mechanistic understanding of how repeated drought–rewetting cycles alter the turnover of different SOC pools remains limited. Here, our analysis quantifies these effects by linking SOC decomposition and soil water dynamics. Two key results emerge from our analysis:

First, drought increased soil CO_2_ emissions and reduced the size of all four active SOC pools, confirming that both labile (DPM + RPM) and stable (BIO + HUM) fractions were vulnerable under intensified drying. The C use efficiency of fresh organic matter (CUE_I_) was nearly twice that of the more stable pools (CUE_S_). This result highlights that, in wet heathland systems dominated by *Calluna vulgaris* , fresh organic matter entering the soil was rapidly decomposed and stabilised in soil. Our findings also support the hypothesis that, given the limited capacity of organo‐mineral soils to buffer drying, severe droughts in heathland ecosystems destabilise nutrient‐rich organic horizons (Sowerby et al. 2008). In our simulations, changes in CSE varied systematically with soil water and drought frequency, revealing a reversal in CSE sensitivity to moisture. During frequent droughts, CSE remained near pre‐drought levels, whereas under prolonged droughts CSE was suppressed. At moderate soil water conditions, CSE increased progressively with shorter drought intervals (12 > 24 > 48 > 96 years) but declined under both low and high soil moisture. Our results align with experimental evidence showing that frequent drought perturbations maintain rapid substrate recycling, whereas long droughts drive systems toward net mineralisation (Müller and Bahn 2022).

Although humification in ECOSSE is not intrinsically depth dependent and the simulated profile was only 30 cm deep, both HUM and BIO pools declined in drought simulations relative to the control with increasing soil depth (Figure 3; Table S4). The largest reductions in HUM occurred between 10 and 25 cm depth, where C stabilisation dominates, while BIO declined almost uniformly with depth. Thus, frequent droughts limited microbial recovery and humus formation, whereas longer droughts intensified the decomposition of HUM in deeper layers. Similar depth‐dependent responses were observed experimentally, where microbial relocation to wetter microsites during drying accelerates the turnover of older C upon rewetting (Allison 2023; Heckman et al. 2023).

The second main result is linked to the sensitivity of CSE to soil water differed between drought and post‐drought periods. The relationship was positive during drought and negative after rewetting. This pattern outlines a pool‐state imbalance that persisted after the termination of drought, a structural reorganisation of C routing that extended beyond hydrological recovery. In ECOSSE, this emerged from a reduced BIO‐HUM transfer efficiency, here quantified by the stabilisation efficiency (SE), which revealed a loss of C‐stabilisation potential between drought simulation phases. Frequent drought‐rewetting cycles therefore sustained higher short‐term SE but weakened long‐term C stabilisation by altering the relative size and coupling of BIO and HUM pools.

Overall, our results demonstrate that drought frequency, rather than the intensity of individual events, governed the stability of soil C pools. This conclusion agrees with recent studies showing that repeated droughts reduced microbial CUE and weakened C stabilisation, driving soils from efficient SOM recycling toward net mineralisation (Cordero et al. 2023; Canarini et al. 2021). In our model, this transition represented a persistent reorganisation of SOC pools, reinforcing the mechanistic link between drought and carbon imbalance in organo‐mineral soil (Canarini et al. 2017).

### 4.2 Modelling Drought Effects on Soil Carbon Dynamics

There is still a paucity of information on modelling the long‐term dynamics of soil C when exposed to drought (Allison 2023). To date, most modelling studies have focused on describing the processes underlying short‐term drying–rewetting events (pulse events) and their effects on microbial respiration, microbial biomass C, and dissolved organic matter (Waring and Powers 2016; Zhou et al. 2021). Long‐term drought experiments have instead concentrated on the impact of drought on microbial CUE, reporting changes in microbial biomass and community composition (Canarini et al. 2021; Green et al. 2019; Huang et al. 2021). By extending a validated 6‐year climate sequence over multiple decades, our modelling approach aimed to combine the benefits of both short‐ and long‐term experiments, linking short‐term processes to emergent, frequency‐dependent dynamics. In particular, our model outputs successfully reproduced the observed magnitudes of soil C loss observed at the experimental site (Domínguez et al. 2015). After 10 years of simulated drought, total SOC declined by approximately 1.8% ± 0.6% (~1820 kg C ha^−1^), with 87% of the losses from RPM and 7% from HUM compared to control. These reductions were lower than the 26% decrease reported by Gliesch et al. (2024) in low‐C heathlands, but the pool‐specific contributions followed the same order.

The pool‐state imbalances discussed in the previous section can be viewed through the lens of emerging modelling frameworks that link microbial processing and mineral stabilisation in the formation of mineral‐associated organic matter (MAOM) (Tao et al. (2024) and references therein). These frameworks emphasise that soil C stabilisation arises from microbial transformation of substrates and their subsequent association with minerals, governed by sorption–desorption kinetics and mineral surface properties. In contrast, ECOSSE represents a conventional compartment model in which C stabilisation is described as first‐order transfers between conceptual SOC pools rather than through explicit mineral interactions in measurable fractions. Within this simplified structure, the observed decline in SE during post‐drought phases can be interpreted as a macro‐scale analogue of the microbial‐mineral decoupling proposed by MAOM theory. To this end, while ECOSSE cannot reproduce the molecular‐level mechanisms of MAOM formation, our results outlined that its mechanistic framework captured a loss of stabilisation efficiency under repeated drought, consistent with the direction of more recent process‐based soil modelling.

### 4.3 Modelling Limitations

In our study, we used a 6‐year sequence (2009–2014) of observed climate and soil respiration data to project decadal trends. This pragmatic modelling approach allowed us to extend our short‐term field observations, but in doing so imposed several model constraints. The repeated sequence removed interannual variability and excluded directional climatic trends, preventing realistic wet–dry sequencing and long‐term climatic drift (Chen et al. 2012; Wieder et al. 2021). Consequently, in our simulations the SOC pools could not fully re‐equilibrate beyond the repeated cycle, and recovery trajectories may have appeared more gradual and less variable than they would under natural climatic fluctuations. Moreover, ECOSSE does not explicit simulate microbial processes: enzyme kinetics, dormancy, or acclimation processes are not included, which are incorporated in newer microbial‐explicit models (Chandel et al. 2023; Schwarz et al. 2024; Wieder et al. 2015). To this end, the model could not account for stochastic variability or adaptive feedback processes. Despite these simplifications, our simulations showed persistent changes in the relative sizes of the SOC pools, where changes initiated by drought continued to influence decomposition and stabilisation after rewetting. These effects arose from the structural formulation of ECOSSE, in which the amount of C held in each pool governs subsequent fluxes, rather than from any explicit representation of ecological feedback processes or legacy effects.

An additional limitation of our modelling approach was the use of fixed seasonal Rh/Rs fractions to partition total soil respiration (Rs) into heterotrophic (Rh) and autotrophic (Ra) components. This pragmatic assumption, common when continuous field partitioning is infeasible, necessarily simplified the temporal coupling between soil moisture, temperature, and biological activity. In that respect, in organo‐mineral heathland soils, drought events were reported to reduce microbial activity and substrate diffusion, reducing Rh relative to its assumed fraction (Emmett et al. 2004; Sowerby et al. 2008; Robinson et al. 2016; Seaton et al. 2022). Upon rewetting, microbial respiration often increased temporarily through “Birch‐type” pulses (Canarini et al. 2017), again diverging from the fixed seasonal pattern. These findings imply that using constant seasonal fractions may have obscured transient fluctuations in Rh/Rs during drought–rewetting transitions.

## 5 Conclusions

While drought events are known to accelerate the decomposition of soil organic matter (SOM) and increase soil CO_2_ emissions, it remains unclear whether there is a threshold in drought frequency and intensity that can trigger persistent changes in the capacity of ecosystems to sequester soil C. In this study, we addressed this question using experimental data from a wet heathland system on organo‐mineral soil, coupled with the ECOSSE biogeochemical model, to evaluate how recurrent droughts influence long‐term C stabilisation efficiency. We found that with increasing drought frequency, soil C sequestration efficiency (CSE) decreased, triggering a shift in the relationship between C sequestration and soil water availability. These results demonstrated that drought frequency, rather than the intensity of individual events, played the dominant role in shaping CSE. As droughts became more frequent, the relationship between CSE and soil water reversed: moderate moisture promoted higher efficiency under short drought intervals, whereas prolonged drying reduced CSE even after soil moisture returned to optimal levels. Under prolonged soil water changes, decomposition of stable SOC pools occurred deeper in the profile and even at moisture levels above field capacity. Depth‐resolved analyses revealed that these changes were associated with sustained reductions in microbial biomass (BIO) and humified organic matter (HUM) pools throughout the soil column. Recurrent droughts therefore not only suppressed microbial recovery but also constrained the formation and persistence of stable C in deeper horizons. By partitioning C use efficiency into its labile and stable components (CUE_I_ and CUE_S_) and analysing their co‐variation with soil water, we identified a persistent imbalance in the routing of C between microbial processing and stabilisation. This imbalance, quantified through a reduction in the stabilisation‐efficiency index (SE), reflected a structural decoupling between BIO and HUM pools that persisted after rewetting, once drought events had ceased.

The approach developed here provides a diagnostic framework for quantifying drought impacts on SOC pools using conventional compartmental soil models. Although ECOSSE does not include explicit microbial acclimation, enzyme kinetics, or mineral‐association processes, its simulations reproduced a sustained decline in the efficiency of microbial‐C stabilisation following repeated droughts. Future research should extend this framework to models that explicitly incorporate microbial physiology and organo‐mineral interactions, thereby improving predictions of soil C resilience under intensifying climate change.

## Author Contributions

**Fabrizio Albanito:** conceptualization, formal analysis, writing – original draft, writing – review and editing. **Sabine Reinsch:** data curation, writing – review and editing. **Mark Richards:** supervision. **Amanda M. Thomson:** funding acquisition, writing – review and editing. **Bernard J. Cosby:** writing – review and editing. **Bridget A. Emmett:** writing – review and editing. **David A. Robinson:** conceptualization, writing – review and editing.

## Conflicts of Interest

The authors declare no conflicts of interest.

## Supporting information


**Appendix S1:** gcb70657‐sup‐0001‐AppendixS1.docx.

## Data Availability

The data that support the findings of this study are openly available through the NERC Environmental Information Data Centre repository. The DOI is https://doi.org/10.5285/a81c6520‐c116‐4e99‐810d‐23eba79b9b3f.

## References

[gcb70657-bib-0001] Aghakouchak, A. , A. Mirchi , K. Madani , et al. 2021. “Anthropogenic Drought: Definition, Challenges, and Opportunities.” Reviews of Geophysics 59: e2019RG000683.

[gcb70657-bib-0002] Allison, S. D. 2023. “Microbial Drought Resistance May Destabilize Soil Carbon.” Trends in Microbiology 31: 780–787. 37059647 10.1016/j.tim.2023.03.002

[gcb70657-bib-0003] Bai, Y. , T. Wagener , and P. Reed . 2009. “A Top‐Down Framework for Watershed Model Evaluation and Selection Under Uncertainty.” Environmental Modelling & Software 24: 901–916.

[gcb70657-bib-0004] Canarini, A. , L. P. Kiær , and F. A. Dijkstra . 2017. “Soil Carbon Loss Regulated by Drought Intensity and Available Substrate: A Meta‐Analysis.” Soil Biology & Biochemistry 112: 90–99.

[gcb70657-bib-0005] Canarini, A. , H. Schmidt , L. Fuchslueger , et al. 2021. “Ecological Memory of Recurrent Drought Modifies Soil Processes via Changes in Soil Microbial Community.” Nature Communications 12: 5308. 10.1038/s41467-021-25675-4 PMC8421443 34489463

[gcb70657-bib-0006] Cardozo, G. A. , F. Volaire , P. Chapon , C. Barotin , and K. Barkaoui . 2024. “Can We Identify Tipping Points of Resilience Loss in Mediterranean Rangelands Under Increased Summer Drought?” Ecology 105: e4383. 39054896 10.1002/ecy.4383

[gcb70657-bib-0007] Chandel, A. K. , L. Jiang , and Y. Luo . 2023. “Microbial Models for Simulating Soil Carbon Dynamics: A Review.” Journal of Geophysical Research: Biogeosciences 128: e2023JG007436.

[gcb70657-bib-0008] Chen, S. , Y. Huang , J. Zou , et al. 2012. “Interannual Variability in Soil Respiration From Terrestrial Ecosystems in China and Its Response to Climate Change.” Science China Earth Sciences 55: 2091–2098.

[gcb70657-bib-0009] Cleveland, C. C. , W. R. Wieder , S. C. Reed , and A. R. Townsend . 2010. “Experimental Drought in a Tropical Rain Forest Increases Soil Carbon Dioxide Losses to the Atmosphere.” Ecology 91: 2313–2323. 20836453 10.1890/09-1582.1

[gcb70657-bib-0010] Cordero, I. , A. Leizeaga , L. C. Hicks , J. Rousk , and R. D. Bardgett . 2023. “High Intensity Perturbations Induce an Abrupt Shift in Soil Microbial State.” ISME Journal 17: 2190–2199. 37814127 10.1038/s41396-023-01512-y PMC10690886

[gcb70657-bib-0011] Correndo, A. A. , L. H. M. Rosso , C. H. Hernandez , et al. 2022. “Metrica: An R Package to Evaluate Prediction Performance of Regression and Classification Point‐Forecast Models.” Journal of Open Source Software 7: 4655.

[gcb70657-bib-0012] Costa, F. R. C. , J. Schietti , S. C. Stark , and M. N. Smith . 2023. “The Other Side of Tropical Forest Drought: Do Shallow Water Table Regions of Amazonia Act as Large‐Scale Hydrological Refugia From Drought?” New Phytologist 237: 714–733. 35037253 10.1111/nph.17914

[gcb70657-bib-0013] Deng, L. , C. Peng , D.‐G. Kim , et al. 2021. “Drought Effects on Soil Carbon and Nitrogen Dynamics in Global Natural Ecosystems.” Earth‐Science Reviews 214: 103501.

[gcb70657-bib-0014] Domínguez, M. T. , A. R. Smith , S. Reinsch , and B. A. Emmett . 2017. “Inter‐Annual Variability of Soil Respiration in Wet Shrublands: Do Plants Modulate Its Sensitivity to Climate?” Ecosystems 20: 796–812.

[gcb70657-bib-0015] Domínguez, M. T. , A. Sowerby , A. R. Smith , et al. 2015. “Sustained Impact of Drought on Wet Shrublands Mediated by Soil Physical Changes.” Biogeochemistry 122: 151–163.

[gcb70657-bib-0016] Dong, H. X. , S. Zhang , J. J. Lin , and B. Zhu . 2021. “Responses of Soil Microbial Biomass Carbon and Dissolved Organic Carbon to Drying‐Rewetting Cycles: An Analysis.” Catena 207: 105610.

[gcb70657-bib-0017] Emmett, B. A. , C. Beier , M. Estiarte , et al. 2004. “The Response of Soil Processes to Climate Change: Results From Manipulation Studies of Shrublands Across an Environmental Gradient.” Ecosystems 7: 625–637.

[gcb70657-bib-0018] Evans, C. D. , M. Peacock , A. J. Baird , et al. 2021. “Overriding Water Table Control on Managed Peatland Greenhouse Gas Emissions.” Nature 593: 548–552. 33882562 10.1038/s41586-021-03523-1

[gcb70657-bib-0019] Gliesch, M. , L. H. Sanchez , E. Jongepier , et al. 2024. “Heathland Management Affects Soil Response to Drought.” Journal of Applied Ecology 61: 1372–1384.

[gcb70657-bib-0020] Gottschalk, P. , J. Bellarby , C. Chenu , et al. 2010. “Simulation of Soil Organic Carbon Response at Forest Cultivation Sequences Using C‐13 Measurements.” Organic Geochemistry 41: 41–54.

[gcb70657-bib-0021] Green, J. K. , S. I. Seneviratne , A. M. Berg , et al. 2019. “Large Influence of Soil Moisture on Long‐Term Terrestrial Carbon Uptake.” Nature 565: 476–479. 30675043 10.1038/s41586-018-0848-x PMC6355256

[gcb70657-bib-0022] Hallett, S. H. , R. Sakrabani , C. A. Keay , and J. A. Hannam . 2017. “Developments in Land Information Systems: Examples Demonstrating Land Resource Management Capabilities and Options.” Soil Use and Management 33: 514–529.

[gcb70657-bib-0023] Hastings, A. F. , M. Wattenbach , W. Eugster , C. S. Li , N. Buchmann , and P. Smith . 2010. “Uncertainty Propagation in Soil Greenhouse Gas Emission Models: An Experiment Using the DNDC Model and at the Oensingen Cropland Site.” Agriculture, Ecosystems & Environment 136: 97–110.

[gcb70657-bib-0024] He, X. , E. Abs , S. D. Allison , et al. 2024. “Emerging Multiscale Insights on Microbial Carbon Use Efficiency in the Land Carbon Cycle.” Nature Communications 15: 8010. 10.1038/s41467-024-52160-5 PMC11399347 39271672

[gcb70657-bib-0025] Heckman, K. A. , A. R. Possinger , B. D. Badgley , et al. 2023. “Moisture‐Driven Divergence in Mineral‐Associated Soil Carbon Persistence.” Proceedings of the National Academy of Sciences 120: 146830. 10.1073/pnas.2210044120 PMC9962923 36745807

[gcb70657-bib-0026] Huang, H. , S. Calabrese , and I. Rodriguez‐Iturbe . 2021. “Variability of Ecosystem Carbon Source From Microbial Respiration Is Controlled by Rainfall Dynamics.” Proceedings of the National Academy of Sciences of the United States of America 118: e2115283118. 34930848 10.1073/pnas.2115283118 PMC8719902

[gcb70657-bib-0027] Huxman, T. E. , M. D. Smith , P. A. Fay , et al. 2004. “Convergence Across Biomes to a Common Rain‐Use Efficiency.” Nature 429: 651–654. 15190350 10.1038/nature02561

[gcb70657-bib-0028] Knapp, A. K. , K. V. Condon , C. C. Folks , et al. 2024. “Field Experiments Have Enhanced Our Understanding of Drought Impacts on Terrestrial Ecosystems‐But Where Do We Go From Here?” Functional Ecology 38: 76–97.

[gcb70657-bib-0029] Kopittke, G. R. , A. Tietema , E. E. van Loon , and K. Kalbitz . 2013. “The Age of Managed Heathland Communities: Implications for Carbon Storage?” Plant and Soil 369: 219–230.

[gcb70657-bib-0030] Kopittke, G. R. , E. E. van Loon , A. Tietema , and D. Asscheman . 2013. “Soil Respiration on an Aging Managed Heathland: Identifying an Appropriate Empirical Model for Predictive Purposes.” Biogeosciences 10: 3007–3038.

[gcb70657-bib-0031] Liu, L. , M. Estiarte , P. Bengtson , et al. 2022. “Drought Legacies on Soil Respiration and Microbial Community in a Mediterranean Forest Soil Under Different Soil Moisture and Carbon Inputs.” Geoderma 405: 115425.

[gcb70657-bib-0032] Masuda, N. , K. Aihara , and N. G. Maclaren . 2024. “Anticipating Regime Shifts by Mixing Early Warning Signals From Different Nodes.” Nature Communications 15: 1086. 10.1038/s41467-024-45476-9 PMC10844243 38316802

[gcb70657-bib-0033] Maurer, G. E. , A. J. Hallmark , R. F. Brown , O. E. Sala , and S. L. Collins . 2020. “Sensitivity of Primary Production to Precipitation Across the United States.” Ecology Letters 23: 527–536. 31912647 10.1111/ele.13455

[gcb70657-bib-0034] Müller, L. M. , and M. Bahn . 2022. “Drought Legacies and Ecosystem Responses to Subsequent Drought.” Global Change Biology 28: 5086–5103. 35607942 10.1111/gcb.16270 PMC9542112

[gcb70657-bib-0035] Reinsch, S. , E. Koller , A. Sowerby , et al. 2017. “Shrubland Primary Production and Soil Respiration Diverge Along European Climate Gradient.” Scientific Reports 7: 43952. 28256623 10.1038/srep43952 PMC5335567

[gcb70657-bib-0036] Reinsch, S. , D. A. Robinson , M. A. J. Van Soest , A. M. Keith , S. Parry , and A. M. Tye . 2024. “Temperate Soils Exposed to Drought—Key Processes, Impacts, Indicators, and Unknowns.” Land 13: 1759.

[gcb70657-bib-0037] Reinsch, S. , A. Sowerby , and B. A. Emmett . 2015a. Daily Automated Weather Station (AWS) Data From Climoor Fieldsite in Clocaenog Forest 1999–2015. NERC Environmental Information Data Centre.

[gcb70657-bib-0038] Reinsch, S. , A. Sowerby , and B. A. Emmett . 2015b. Daily Plot Level (Micro Meteorological) Data at Climoor Field Site in Clocaenog Forest 1998–2015. NERC Environmental Information Data Centre.

[gcb70657-bib-0039] Reinsch, S. , A. Sowerby , and B. A. Emmett . 2015c. Fortnightly Soil Respiration Data From Climoor Fieldsite in Clocaenog Forest 1999–2015. NERC Environmental Information Data Centre.

[gcb70657-bib-0040] Reinsch, S. , A. Sowerby , and B. A. Emmett . 2015d. Litterfall Data From Climoor Fieldsite in Clocaenog Forest 1999–2011. NERC Environmental Information Data Centre.

[gcb70657-bib-0065] Robinson, D. , S. Jones , I. Lebron , et al. 2016. “Experimental Evidence for Drought Induced Alternative Stable States of Soil Moisture.” Scientific Reports 6: 20018. 10.1038/srep20018. 26804897 PMC4726285

[gcb70657-bib-0041] Robinson, D. A. , J. W. Hopmans , V. Filipovic , et al. 2019. “Global Environmental Changes Impact Soil Hydraulic Functions Through Biophysical Feedbacks.” Global Change Biology 25: 1895–1904. 30900360 10.1111/gcb.14626

[gcb70657-bib-0042] Schrumpf, M. , K. Kaiser , G. Guggenberger , T. Persson , I. Kögel‐Knabner , and E. D. Schulze . 2013. “Storage and Stability of Organic Carbon in Soils as Related to Depth, Occlusion Within Aggregates, and Attachment to Minerals.” Biogeosciences 10: 1675–1691.

[gcb70657-bib-0043] Schwarz, E. , S. Ghersheen , S. Belyazid , and S. Manzoni . 2024. “When and Why Microbial‐Explicit Soil Organic Carbon Models Can Be Unstable.” Biogeosciences 21: 3441–3461.

[gcb70657-bib-0044] Seaton, F. M. , S. Reinsch , T. Goodall , et al. 2022. “Long‐Term Drought and Warming Alter Soil Bacterial and Fungal Communities in an Upland Heathland.” Ecosystems 25: 1279–1294.

[gcb70657-bib-0045] Sierra, C. A. , S. E. Trumbore , E. A. Davidson , S. Vicca , and I. Janssens . 2015. “Sensitivity of Decomposition Rates of Soil Organic Matter With Respect to Simultaneous Changes in Temperature and Moisture.” Journal of Advances in Modeling Earth Systems 7: 335–356.

[gcb70657-bib-0046] Smith, C. S. , M. E. Rudd , R. K. Gittman , et al. 2020. “Coming to Terms With Living Shorelines: A Scoping Review of Novel Restoration Strategies for Shoreline Protection.” Frontiers in Marine Science 7: 434.

[gcb70657-bib-0047] Smith, J. , P. Gottschalk , J. Bellarby , et al. 2010a. “Estimating Changes in Scottish Soil Carbon Stocks Using ECOSSE. I. Model Description and Uncertainties.” Climate Research 45: 179–192.

[gcb70657-bib-0048] Smith, J. , P. Gottschalk , J. Bellarby , et al. 2010b. “Estimating Changes in Scottish Soil Carbon Stocks Using ECOSSE. II. Application.” Climate Research 45: 193–205.

[gcb70657-bib-0049] Sowerby, A. , B. A. Emmett , A. Tietema , and C. Beier . 2008. “Contrasting Effects of Repeated Summer Drought on Soil Carbon Efflux in Hydric and Mesic Heathland Soils.” Global Change Biology 14: 2388–2404.

[gcb70657-bib-0050] Sowerby, A. , B. A. Emmett , D. Williams , C. Beier , and C. D. Evans . 2010. “The Response of Dissolved Organic Carbon (DOC) and the Ecosystem Carbon Balance to Experimental Drought in a Temperate Shrubland.” European Journal of Soil Science 61: 697–709.

[gcb70657-bib-0051] Stovícek, A. , M. Kim , D. Or , and O. Gillor . 2017. “Microbial Community Response to Hydration‐Desiccation Cycles in Desert Soil.” Scientific Reports 7: 45735. 28383531 10.1038/srep45735 PMC5382909

[gcb70657-bib-0052] Tao, F. , B. Z. Houlton , Y. Y. Huang , et al. 2024. “Convergence in Simulating Global Soil Organic Carbon by Structurally Different Models After Data Assimilation.” Global Change Biology 30: e17297. 38738805 10.1111/gcb.17297

[gcb70657-bib-0053] Tao, F. , Y. Huang , B. A. Hungate , et al. 2023. “Microbial Carbon Use Efficiency Promotes Global Soil Carbon Storage.” Nature 618: 981–985. 37225998 10.1038/s41586-023-06042-3 PMC10307633

[gcb70657-bib-0054] Thornthwaite, C. W. 1948. “An Approach Toward a Rational Classification of Climate.” Soil Science 66: 55–94.

[gcb70657-bib-0055] Vahedifard, F. , C. C. Goodman , V. Paul , and A. Aghakouchak . 2024. “Amplifying Feedback Loop Between Drought, Soil Desiccation Cracking, and Greenhouse Gas Emissions.” Environmental Research Letters 19: 031005.

[gcb70657-bib-0056] Vicente‐Serrano, S. M. , S. Beguería , and J. I. López‐Moreno . 2010. “A Multiscalar Drought Index Sensitive to Global Warming: The Standardized Precipitation Evapotranspiration Index.” Journal of Climate 23: 1696–1718.

[gcb70657-bib-0057] Waring, B. G. , and J. S. Powers . 2016. “Unraveling the Mechanisms Underlying Pulse Dynamics of Soil Respiration in Tropical Dry Forests.” Environmental Research Letters 11: 105005.

[gcb70657-bib-0058] Wieder, W. R. , S. D. Allison , E. A. Davidson , et al. 2015. “Explicitly Representing Soil Microbial Processes in Earth System Models.” Global Biogeochemical Cycles 29: 1782–1800.

[gcb70657-bib-0059] Wieder, W. R. , Z. Butterfield , K. Lindsay , D. L. Lombardozzi , and G. Keppel‐Aleks . 2021. “Interannual and Seasonal Drivers of Carbon Cycle Variability Represented by the Community Earth System Model (CESM2).” Global Biogeochemical Cycles 35: e2021GB007034. 10.1029/2021GB007034 PMC9285408 35860341

[gcb70657-bib-0060] Wu, L. , Y. Zhang , X. Guo , et al. 2022. “Reduction of Microbial Diversity in Grassland Soil Is Driven by Long‐Term Climate Warming.” Nature Microbiology 7: 1054–1062. 10.1038/s41564-022-01147-3 35697795

[gcb70657-bib-0061] Wunderling, N. , A. Staal , B. Sakschewski , et al. 2022. “Recurrent Droughts Increase Risk of Cascading Tipping Events by Outpacing Adaptive Capacities in the Amazon Rainforest.” Proceedings of the National Academy of Sciences of the United States of America 119: e2120777119. 35917341 10.1073/pnas.2120777119 PMC9371734

[gcb70657-bib-0062] Zhong, Y. , M. Jiang , and B. A. Middleton . 2020. “Effects of Water Level Alteration on Carbon Cycling in Peatlands.” Ecosystem Health and Sustainability 6: 1806113.

[gcb70657-bib-0063] Zhou, J. , S. P. Chen , L. M. Yan , et al. 2021. “A Comparison of Linear Conventional and Nonlinear Microbial Models for Simulating Pulse Dynamics of Soil Heterotrophic Respiration in a Semi‐Arid Grassland.” Journal of Geophysical Research – Biogeosciences 126: e2020JG006120.

[gcb70657-bib-0064] Zhou, Z. , M. J. Liang , L. Chen , et al. 2022. “Processes, Feedbacks, and Morphodynamic Evolution of Tidal Flat‐Marsh Systems: Progress and Challenges.” Water Science and Engineering 15: 89–102.

