# Supplementary material for: Frequent Droughts Reduce Carbon Stabilisation in Organo‐Mineral Soils
Source: Glob Chang Biol. 2026 Jan 6;32(1):e70657. doi: 10.1111/gcb.70657 (PMC12771270; doi:10.1111/gcb.70657)
Supplement: Supplementary file 1 — Appendix S1: gcb70657‐sup‐0001‐AppendixS1.docx. [file GCB-32-e70657-s001.docx]

SUPPLEMENTARY INFORMATION

Frequent droughts reduce carbon stabilisation in ORGANO-MINERAL SOILS

Fabrizio Albanito^1*^, Sabine Reinsch^1^, Mark Richards^2^, Amanda M. Thomson^1^, Bernard J. Cosby^1^, Bridget A. Emmett^1^, and David A. Robinson^1^

*^1^UK Centre for Ecology & Hydrology, Deiniol Road, Bangor, LL57 2UW, UK.*

*^2^Institute of Biological and Environmental Sciences, University of Aberdeen, Aberdeen, UK*

Text S1: ECOSSE model and theoretical context

ECOSSE is classified as a decomposition model which includes the following five basic principles: 1) mass balance, 2) substrate dependence, 3) heterogeneity of decomposition rates across different soil organic carbon (SOC) compartments, 4) transformations of soil organic matter (SOM), and 5) environmental variability effects(Manzoni and Porporato, 2009; Sierra and Müller, 2015). These five principles are applied across different soil profile layers connected through the water flow, which drives the advection of organic and inorganic dissolved compounds along the profile.

The model estimates annual net primary productivity (NPP) using the MIAMI model (Lieth, 1975). In the model NPP is calculated twice, once based on the long-term mean air temperature (TA) and the second based on precipitation (P). The lower of the two NPP estimations is the net primary production (NPP) used in the simulations of SOC dynamics (i.e. whichever climate variable is the limiting factor). The annual plant C inputs to the soil is calculated as a fixed fraction of NPP to account for differences in the proportion of C lost during the year (due to grazing or plant mortality). In the model, SOC is split into four active compartments and a small amount of inert organic matter (IOM), which is assumed to not contribute to the decomposition processes. The four active compartments are Decomposable Plant Material (DPM), Resistant Plant Material (RPM), Microbial Biomass (BIO, or active organic matter) and Humified Organic Matter (HUM). All incoming plant inputs enter the soil as RPM and DPM, and over time decompose to form carbon dioxide (CO_2_), BIO and HUM. The BIO + HUM pool is then split into 46% BIO and 54% HUM. Organic matter (OM) is exchanged between the four SOC pools according to first-order kinetics, characterized by a specific rate constant for each pool, and modified according to rate modifiers dependent on the temperature, water content, plant cover and pH of the soil.

The ratio of decomposable and resistant plant material (DPM/RPM) is used directly in the model to allow for changes in the ‘quality’ or decomposability of the input from vegetation to the soil. Based on the high lignin content characterizing the woody tissues of heathland vegetation (Hopkins et al., 1988), we assumed that the plant input produced at the experimental site has an initial DPM/RPM ratio of 0.25 (i.e. 20% is DPM and 80% is RPM). Over time BIO and HUM decompose to form more CO_2_, BIO and HUM. The impact of soil texture on SOM decomposition is accounted for in the partitioning of decomposing OM into the pools BIO and HUM pools by simulating the effect of clay minerals on the physical protection of SOM. In aerobic conditions (i.e. above field capacity), the decomposition process results in gaseous losses of CO_2_. While in anaerobic conditions (i.e. above field capacity), methane (CH_4_) losses start and become significant towards saturated soil moisture conditions. A proportion of the produced CH_4_ is oxidised back to CO_2_ depending on transportation of CH_4_ in plants, the rate of diffusion through the soil and the thickness of the aerobic region in the soil which needs to be crossed by the CH_4_.

ECOSSE uses an iterative procedure to estimate the plant OM inputs and SOC pool sizes from measured soil C. An initial estimate of the total annual OM input is used to provide the first estimation of SOC pool sizes at steady state. We assumed that the SOC pools at the site are in steady state prior to the start of the experimental manipulation. The steady state assumption is valid in a system where no significant disturbance has been observed for an extended period of time, which can be assumed to be true for the upland Heathland system featuring a *Calluna vulgaris* community of 10 to 15 years of age. In this study, this initial modelling phase was run using long-term rainfall and air temperature data at a site level on a monthly time step from 1957 to 2000. After this initial phase, in the forward simulations ECOSSE interactively adjusts the annual OM inputs according to the ratio of simulated to measured soil C, applying changes in the soil conditions and climate to calculate the impact of temporal changes imposed by the drought treatment (and untreated control) on the rate of SOC turnover of the different pools.

To this end, ECOSSE includes rate modifiers to implicitly represent the effects of soil moisture on SOM decomposition and physical processes (Figure S1). At very low level of soil water content (roughly at a soil water tension at -15 bar) SOM decomposition is inhibited due to the water limitation of microbes. Decomposition, however, linearly increases with the increase of soil moisture until optimum conditions for microorganisms are reached at approximately 1 bar. The optimal conditions for SOM decomposition are kept constant until soil water reaches field capacity at approximately -0.05 bar. Above field capacity, SOM decomposition linearly decreases to be completely inhibited at saturation.

Finally, in ECOSSE, soil water availability is calculated in each layer using a "tipping bucket" approach, adapted from the SUNDIAL model (Bradbury et al., 1993; Smith et al., 1997). As precipitation enters the soil, it pushes existing water deeper into the profile. The uppermost soil layer is filled to field capacity first, and any excess precipitation moves downward, filling subsequent layers in the same manner until either all precipitation is used, or the bottom of the profile is reached. Any water remaining after filling all layers to field capacity is divided between drainage (water exiting the soil profile) and excess water, which is used to fill layers to saturation from the bottom of the profile upwards. This process considers the observed water table depth, the water available at saturation, and weather data to determine the fraction of excess water needed to maintain the observed water table depth. Additionally, water is lost from the top of the profile through evapotranspiration, which is estimated using the Thornthwaite method.

**
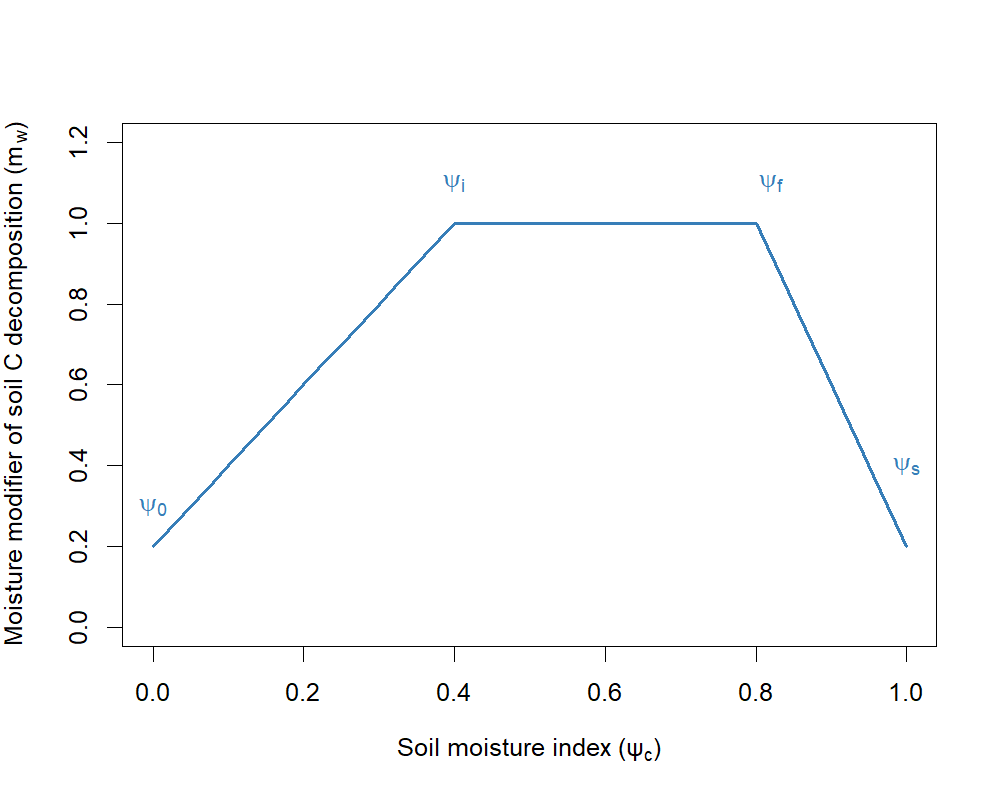
**

Figure S1: Soil moisture modifier (m_w_) used in the ECOSSE model. In the functional forms of moisture modifier (m_w_), decomposition rates are modified for available water content values (ψ_c_) ranging from wilting point (ψ_0_) to field capacity (ψ_f_), and from field capacity to saturated available water levels (ψ_s_). In the Figure, the moisture function was normalized in such a way that the input soil moisture is in a range from 0 to 1. At ψ_0_ (roughly corresponding to a water tension is at -15 bar) soil C decomposition is inhibited, and m_W_ (Eq. S1) has a value of 0.2. Between ψ_0_ and a water tension of -1 bar (ψ_i_) m_W_ linearly increases reaching its maximum value of 1. Above ψ_i_, m_W_ (Eq. S2) is maintained at saturation level until the soil available water content reaches field capacity (ψ_f_) (i.e. a water tension of -0.05 bar). This means that between ψ_i_ and ψ_f_ soil C decomposition is not inhibited as the available soil water content is assumed to be at optimum level for autotrophic and heterotrophic activity (approximately 40% in clay soil). Above optimal soil moisture condition (ψ_f_) a linear reduction is applied to m_W_ which reaches the minimum rate of 0.2 at ψ_s_.

$m_{w}=\frac{\left( 1-m_{w0} \right)\times\left( \psi_{f}-\psi_{c}-\psi_{i} \right)}{\psi_{f}-\psi_{i}};(if \left( \psi_{f}-\psi_{c} \right)> \psi_{i},m_{w}=1)$ (Eq. S1)

$m_{w}=\frac{\left( 1-m_{w0} \right)\times\left( \psi_{c}-\psi_{f} \right)}{\psi_{s}-\psi_{f}}$ (Eq. S2)

Table S1: Monthly water balance (mm) at the Clocaenog site estimated by applying the Thornthwaite method (Thornthwaite, 1948) and the Hydromad R package (Bai et al., 2009) using the median climatic condition (precipitation (P), potential evapotranspiration (PET), and air temperature (TA, °C)) measured during the experimental period from 2009 to 2014. The surplus season occurs when P exceeds potential PET. The utilization season is a time when PET exceeds P. The deficit season occurs when occurs when PET exceeds P. Also see Figure S2.

| Control treatment | | | | | | |
| --- | --- | --- | --- | --- | --- | --- |
|  | **P** | **PET** | **Utilization** | **Surplus** | **TA** | **Deficit** |
| Min. | 53.20 | 12.85 | 1.20 | 75.50 | 12.79 | -6.25 |
| 1st Quartile | 68.25 | 24.35 | 28.43 | 83.87 | 24.35 | -1.35 |
| Median | 103.65 | 40.21 | 62.11 | 92.60 | 38.46 | -0.16 |
| Mean | 104.86 | 37.23 | 68.77 | 88.29 | 36.09 | -1.14 |
| 3rd Quartile | 137.05 | 48.98 | 102.59 | 92.60 | 45.29 | 0.00 |
| Max. | 193.60 | 56.00 | 170.95 | 92.60 | 55.55 | 0.00 |
| Drought treatment | | | | | | |
| Min. | 35.95 | 13.20 | 0 | 68.45 | 13.20 | -12.17 |
| 1st Quartile | 47.93 | 24.37 | 9.75 | 75.40 | 24.12 | -2.87 |
| Median | 74.05 | 40.32 | 28.95 | 84.75 | 39.06 | -0.96 |
| Mean | 70.19 | 37.56 | 35.60 | 82.71 | 34.59 | -2.97 |
| 3rd Quartile | 90.67 | 49.41 | 61.79 | 89.68 | 42.15 | -0.23 |
| Max. | 115.99 | 56.60 | 83.61 | 92.60 | 48.76 | 0.00 |

Table S2: Summary of the above- and below-ground living biomass, mass of litter lying on the soil surface, and changes in ecosystem components observed between the control and drought treatment. Negative values mean that droughts have a negative impact on specific state variables.

|  | Biomass (kg m^-2^) | C content (kg C m^-2^) | Change due to drought treatment (%) |
| --- | --- | --- | --- |
| Calluna vulgaris | 2. 3 (±0.4) | 1.08 (±0.2) | 8 |
| Vaccinium myrtillus | 0.3 (±0.1) | 0.15 (±6.7E-02) | -44 |
| Empetrum nigrum | 6.2E-02 (±3.1E-02) | 2.9E-02(±1.5E-0.2) | 53 |
| Deschampsia flexuosa | 1.1E-02 (±6.1E-03) | 5.1E-0.3 (±2.9E-03) | 42 |
| Mosses | 0.4 (±0.1) | 0.25 (±0.1) | 14 |
| Roots | 3.38 (±0.6) | 1.60 (±0.3) | 7 |
| Litter | 3.2E-03 (±1.7E-0.3) | 1.5E-03 (8.3E-0.4) | 7 |
| **Sum** | **7.33** | **3.48** |  |

Table S3: Sensitivity of modelled (ECOSSE outputs) versus measured monthly heterotrophic respiration (Rh) to uncertainty in the Rh/Rs partitioning. Seasonal Rh/Rs fractions were varied by ±5 % and ±10 % in the drought-treatment data only, while the control data remained fixed at the baseline fractions derived from the literature. Reported metrics (R², RMSE, and MBE) are shown separately for the control and drought treatments.

| Scenario | R² (Control) | RMSE (Control) | MBE (Control) | R² (Drought) | RMSE (Drought) | MBE (Drought) |
| --- | --- | --- | --- | --- | --- | --- |
| Baseline | 0.62 | 1.23 | -0.15 | 0.58 | 1.35 | 0.10 |
| +5% | 0.61 | 1.26 | -0.12 | 0.59 | 1.38 | 0.08 |
| -5% | 0.63 | 1.21 | -0.18 | 0.57 | 1.34 | 0.12 |
| +10% | 0.61 | 1.28 | -0.10 | 0.59 | 1.40 | 0.07 |
| -10% | 0.64 | 1.19 | -0.20 | 0.56 | 1.33 | 0.15 |

Table S4: Impact of drought scenario on soil profile available water (SW, mm), heterotrophic soil respiration (Rh), decomposable plant material (DPM), resistant plant material (RPM), soil biomass (BIO), and humic organic matter (HUM). For each modelling scenario, the SOC values (kg C ha^-1^, mean±sd) correspond to differences between drought and control treatment in the ECOSSE simulations characterized by soil water anomalies. A negative sign corresponds to a decrease compared to the control treatment.

|  | **96-year drought intermittence** | | | | | |
| --- | --- | --- | --- | --- | --- | --- |
| Soil depth (cm) | SW anomaly | Rh | DPM-C | RPM-C | BIO-C | HUM-C |
| 5 | -29.9 (±31.4) | 6.8 (±32.1) | -3.4 (±18.1) | -4.8 (±1.5) | -4.9 (±1.3) | -1.4 (±0.7) |
| 10 | -46.5 (±25.2) | 13.0 (±48.6) | -2.2 (±26.1) | -6.4 (±2.1) | -6.0 (±1.5) | -1.8 (±1.0) |
| 15 | -45.7 (±25.7) | 18.8 (±59.4) | -12.5 (±21.0) | -12.9 (±3.2) | -11.6 (±3.0) | -3.6 (±2.1) |
| 20 | -45.8 (±26.2) | 19.3 (±63.5) | -8.8 (±26.4) | -13.4 (±3.0) | -11.8 (±2.9) | -3.6 (±2.0) |
| 25 | -40.7 (±22.0) | 20.5 (±68.5) | -7.9 (±28.4) | -13.4 (±3.6) | -12.4 (±2.8) | -3.9 (±2.0) |
| 30 | -38.5 (±24.5) | 18.0 (±56.1) | -14.8 (±27.4) | -18.6 (±3.6) | -17.7 (±4.0) | -5.5 (±3.1) |
|  | **48-year drought intermittence** | | | | | |
| 5 | -19.8 (±36.6) | 4.8 (±27.0) | -2.7 (±15.1) | -3.6 (±2.2) | -3.7 (±2.0) | -1.4 (±0.6) |
| 10 | -30.9 (±38.1) | 8.9 (±40.8) | -2.0 (±21.7) | -4.8 (±2.9) | -4.6 (±2.4) | -1.8 (±0.8) |
| 15 | -29.4 (±39.6) | 12.8 (±50.8) | -8.0 (±19.2) | -8.8 (±6.1) | -8.1 (±5.2) | -3.4 (±1.5) |
| 20 | -29.3 (±40.6) | 12.9 (±54.4) | -3.6 (±25.4) | -7.9 (±7.8) | -7.4 (±6.2) | -3.1 (±1.3) |
| 25 | -27.7 (±30.5) | 13.7 (±58.7) | -2.5 (±27.3) | -7.9 (±7.5) | -7.6 (±6.6) | -3.3 (±1.3) |
| 30 | -26.0 (±33.1) | 12.3 (±48.0) | -10.6 (±23.6) | -13.4 (±7.5) | -12.9 (±6.7) | -5.3 (±2.3) |
|  | **24-year drought intermittence** | | | | | |
| 5 | -16.6 (±39.0) | 4.5 (±25.2) | -3.0 (±14.2) | -3.7 (±1.8) | -3.8 (±1.5) | -1.6 (±0.7) |
| 10 | -25.1 (±44.5) | 8.0 (±37.4) | -2.7 (±20.4) | -4.9 (±2.5) | -4.7 (±1.8) | -2.1 (±0.9) |
| 15 | -23.2 (±46.4) | 11.4 (±47.2) | -8.3 (±17.9) | -8.8 (±4.5) | -8.2 (±3.5) | -3.7 (±1.7) |
| 20 | -23.3 (±48.2) | 11.2 (±50.0) | -4.1 (±23.6) | -8.0 (±5.3) | -7.5 (±4.0) | -3.4 (±1.4) |
| 25 | -23.0 (±34.4) | 11.2 (±51.6) | -3.3 (±25.4) | -8.0 (±6.1) | -7.7 (±4.2) | -3.6 (±1.3) |
| 30 | -22.3 (±35.8) | 10.2 (±45.5) | -9.2 (±23.5) | -12.3 (±6.1) | -12.1 (±5.0) | -5.5 (±2.4) |
|  | **12-year drought intermittence** | | | | | |
| 5 | -17.0 (±38.6) | 4.5 (±25.3) | -4.1 (±14.1) | -4.6 (±1.4) | -4.6 (±1.0) | -2.1 (±1.0) |
| 10 | -25.7 (±43.5) | 8.0 (±37.4) | -4.2 (±20.1) | -6.0 (±1.9) | -5.7 (±1.3) | -2.7 (±1.3) |
| 15 | -23.8 (±45.4) | 11.4 (±47.1) | -10.0 (±17.1) | -10.0 (±2.9) | -9.4 (±2.1) | -4.4 (±2.1) |
| 20 | -24.1 (±47.2) | 11.2 (±49.8) | -6.1 (±23.1) | -9.7 (±3.2) | -8.7 (±2.1) | -4.1 (±1.8) |
| 25 | -23.5 (±33.9) | 11.2 (±51.4) | -5.8 (±24.8) | -9.7 (±3.6) | -9.3 (±2.3) | -4.4 (±1.8) |
| 30 | -23.0 (±35.3) | 10.1 (±45.5) | -10.7 (±22.5) | -13.5 (±3.6) | -13.3 (±2.8) | -6.1 (±2.9) |


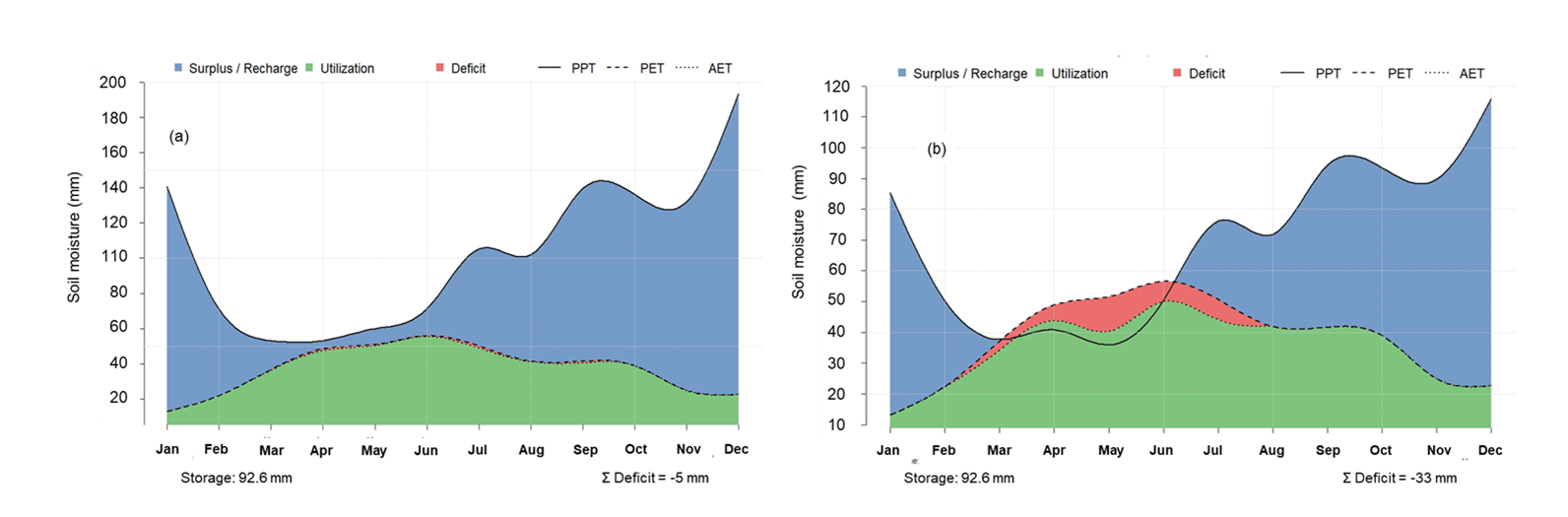


Figure S2: Monthly water balance at the Clocaenog field site estimated using median climatic conditions (precipitation (P, mm), potential evapotranspiration (PET, mm), and air temperature (TA, °C)) measured during the experimental period (2009–2014). (a) Control conditions; (b) Drought conditions. The surplus/recharge season (blue area) occurs when precipitation exceeds PET and the soil has reached field capacity, so additional water contributes to drainage or runoff. The utilization season (green area) occurs when PET exceeds P but soil water availability remains above the wilting point, allowing continued evapotranspiration from stored water. The deficit season (red area) occurs when PET continues to exceed P and soil storage approaches its lower limit, corresponding to the wilting point - that is, when plant-available water has been depleted though residual water persists in the soil matrix. In this framework, zero storage denotes the depletion of plant-available soil water, not the complete absence of soil moisture.


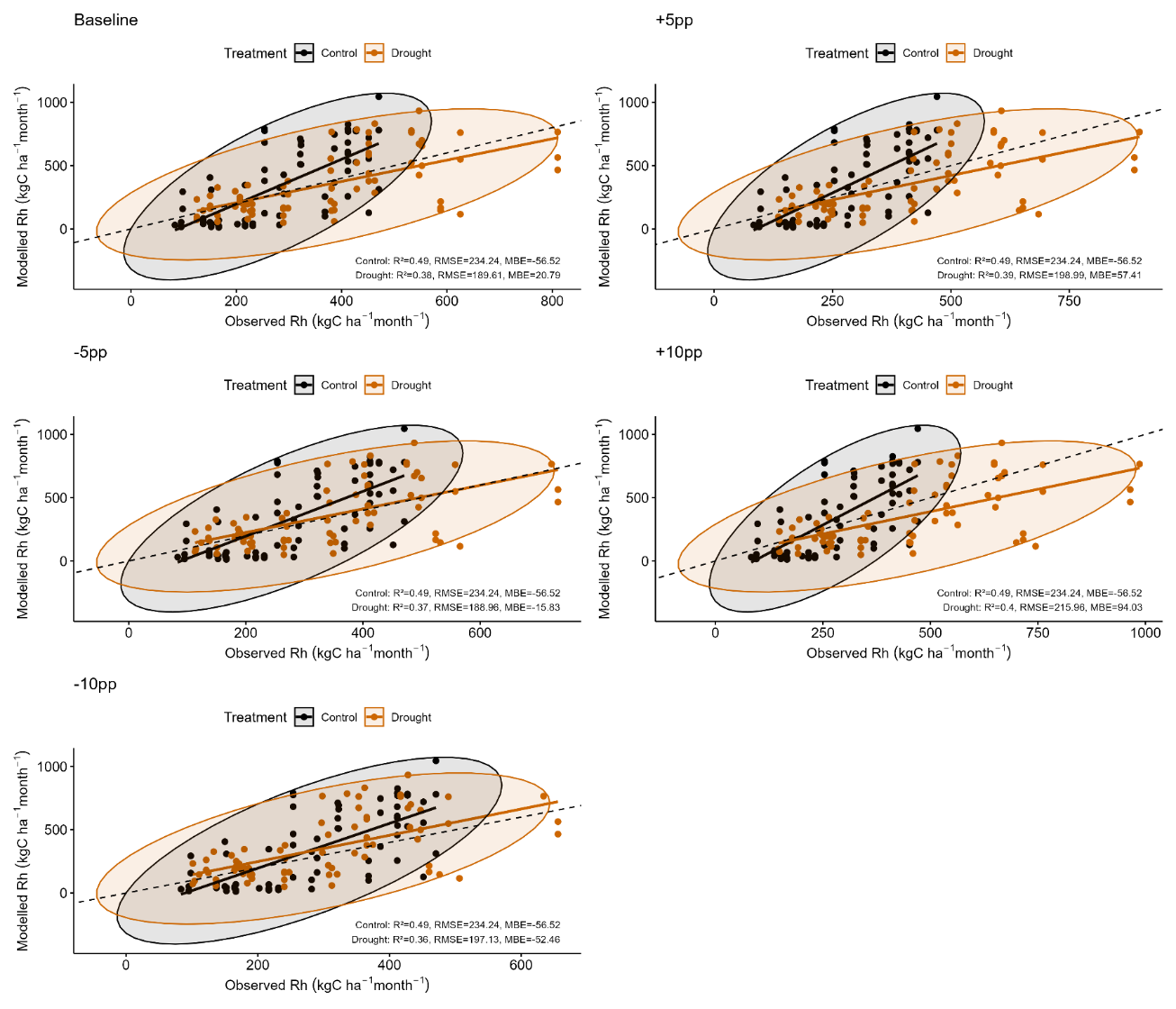


Figure S3: Regression relationship between observed and modelled Rh for 2009-2014 under control (black) and drought (orange) conditions for the baseline and partitioning sensitivity scenarios. For the measured Rs, seasonal Rh/Rs fractions were varied by ±5 and ±10% for the drought treatment series only (control series unchanged). Regression coefficient (R^2^), root mean square error (RMSE) and mean bias error (MBE) of the simulated heterotrophic respiration (Rh, kg CO_2_-C ha^-1^ month^-1^) in the control and drought treatments from 2009 to 2014. Dashed black line is the 1:1 line. The purple and brown shaded areas between the lines are centred at the point representing the sample mean values of the control and drought treatment, respectively. The size and shape of the shaded areas correspond to the 95% confidence interval of observed and modelled Rh.

Table S5: Table A summarize the statistical analyses underlying Figure 4a, which compares Carbon Sequestration Efficiency (CSE) among model scenarios (12‑, 24‑, 48‑, and 96‑years) across soil‑moisture zones (Dry, Moderate, Moist). Kruskal–Wallis tests evaluated overall differences (χ² statistic with 3 degrees of freedom), followed by pairwise Wilcoxon tests (Benjamini–Hochberg adjusted, α = 0.05). The grouping letters indicate which scenarios are not significantly different within each zone (same letter = no difference). Effect sizes (rank-biserial r) quantify the strength of differences, classified as small, moderate, or large. In Table B, Paired Wilcoxon tests comparing each drought scenario against the 96-year reference across matched 10 mm soil-water bins. Δ represents the median CSE difference (scenario − 96-year), and pBH the Benjamini–Hochberg adjusted p-value after controlling for multiple comparisons. pBH < 0.05 means that the difference remains statistically significant even after adjusting for multiple testing. pBH=NA indicates that fewer than three overlapping soil-water bins were available between scenarios, preventing reliable paired statistical comparison (The simulations in 12- and 24-year scenarios may not reach or sustain the same high-water levels as the 96-year baseline).

| **Table A** |  |  |  |  |  |
| --- | --- | --- | --- | --- | --- |
| Moisture zone | | Kruskal-Wallis results | Grouping letters | Effect size | |
| Dry | | χ² = 76.33, p = 1.88×10^-16^ | 12-years=a, 24-years=a, 48-years=b, 96-years=b | 0.29 (moderate) | |
| Moderate | | χ² = 93.16, p = 4.59×10^-20^ | 12-years=a, 24-years=b, 48-years=c, 96-years=d | 0.463 (large) | |
| Moist | | χ² = 27.46, p = 4.71×10^-06^ | 12-years=a, 24-years=a, 48-years=b, 96-years=b | 0.50 (large) | |
| **Table B** | | | | |  |
| Moisture zone | | Significant pairs | Paired_vs_96yrs drought scenario |  |  |
| Dry | | 12-years vs 96-years (p=1.69×10^-13^)  12-years vs 48-years (p=5.61×10^-10^)  24-years vs 96-years (p=1.79×10^-07^)  24-years vs 48-years (p=9.1×10^-06^) | 12-years vs 96-years: Δ=-0.052, pBH=0.151  24-years vs 96-years: Δ=-0.028, pBH=0.151  48-years vs 96-years: Δ=0, pBH=0.855 |  |  |
| Moderate | | 12-years vs 96-years (p=1.04×10^-14^)  24-years vs 96-years (p=3.15×10^-11^)  12-years vs 48-years (p=7.24×10^-10^)  24-years vs 48-years (p=1.01×10^-05^)  12-years vs 24-years (p=0.001)  48-years vs 96-years (p=0.003) | 12-years vs 96-years: Δ=0.171, pBH=0.371  24-years vs 96-years: Δ=0.099, pBH=0.371  48-years vs 96-years: Δ=0.015, pBH=0.371 |  |  |
| Moist | | 12-years vs 96-years (p=7.44×10^-06^)  24-years vs 96-years (p=0.00057)  12-years vs 48-years (p=0.000658)  24-years vs 48-years (p=0.02) | 12-years vs 96-years: Δ=NA, pBH=NA  24-years vs 96-years: Δ=NA, pBH=NA  48-years vs 96-years: Δ=NA, pBH=NA |  |  |

Table S6: Summary of the analysis of covariance (ANCOVA) of regressed CSE on absolute soil water values with a phase interaction to contrast drought vs post‑drought phases across different drought modelling scenarios. The reported drought slope corresponds to the soil water coefficient of the model, and the post‑drought slope is computed as the sum of the drought slope and the soil water × phase (post-drought) interaction term.

| **Component** | **Value** | **Notes** |
| --- | --- | --- |
| ANCOVA slope: drought phase | 0.0001 | Estimated from linear model (CSE ~ soil water * modelling phase); positive slope indicates CSE increases with water during drought periods |
| ANCOVA slope: post-drought phase | -0.0014 | Drought slope + interaction term; negative slope indicates reversal after rewetting stages |
| ANCOVA R^2^ | 0.483 | Overall model fit for ANCOVA model |


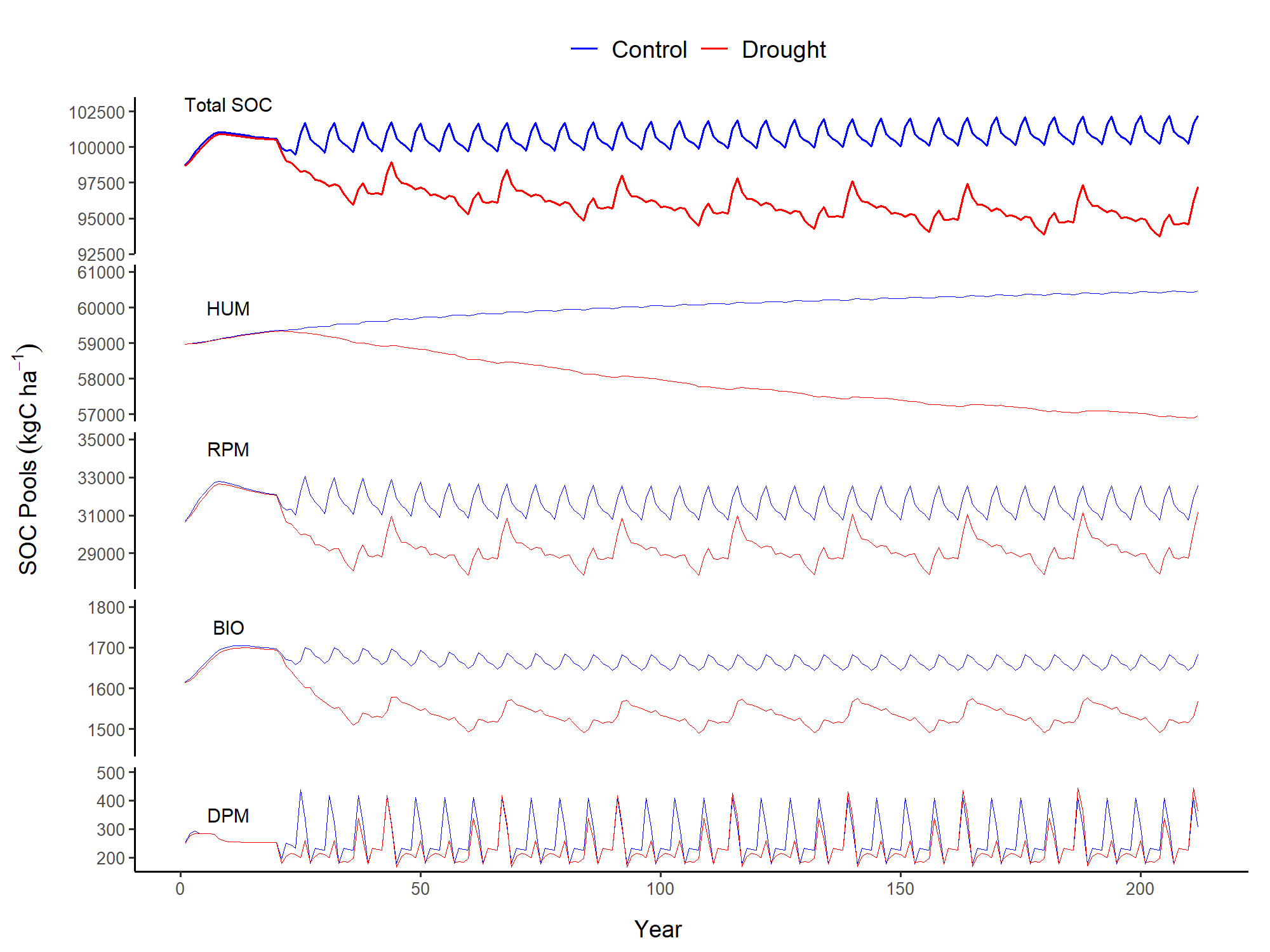


Figure S4: Simulation in the modelling scenario ‘12-year’ in the control and drought treatments of total soil organic carbon (Total SOC) and its corresponding active pools (decomposable plant material (DPM), resistant plant material (RPM), soil biomass (BIO), humic organic matter (HUM).


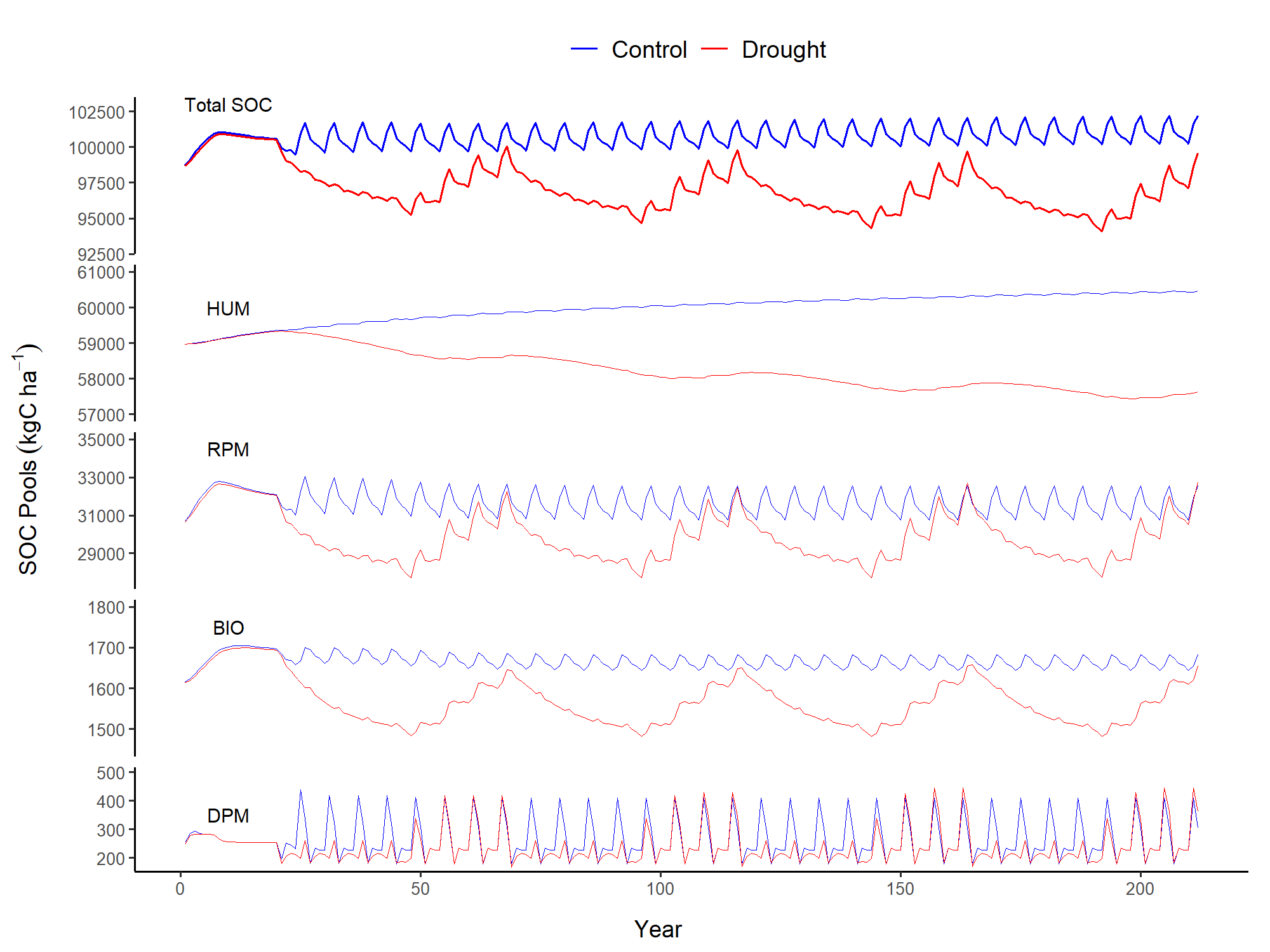


Figure S5: Simulation in the modelling scenario ‘24-year’ in the control and drought treatments of total soil organic carbon (Total SOC) and its corresponding active pools (decomposable plant material (DPM), resistant plant material (RPM), soil biomass (BIO), humic organic matter (HUM).


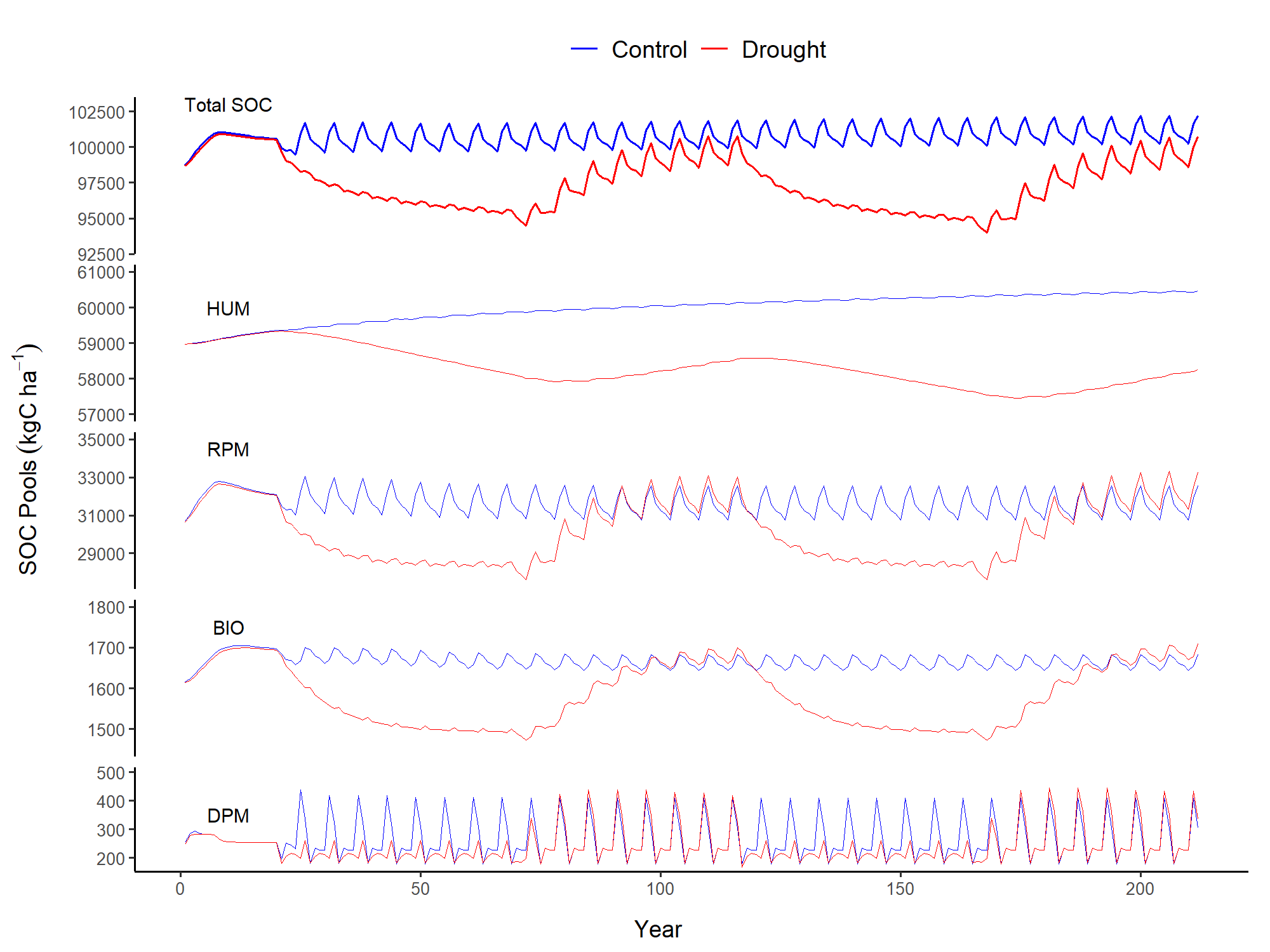


Figure S6: Simulation in the modelling scenario ‘48-year’ in the control and drought treatments of total soil organic carbon (Total SOC) and its corresponding active pools (decomposable plant material (DPM), resistant plant material (RPM), soil biomass (BIO), humic organic matter (HUM).


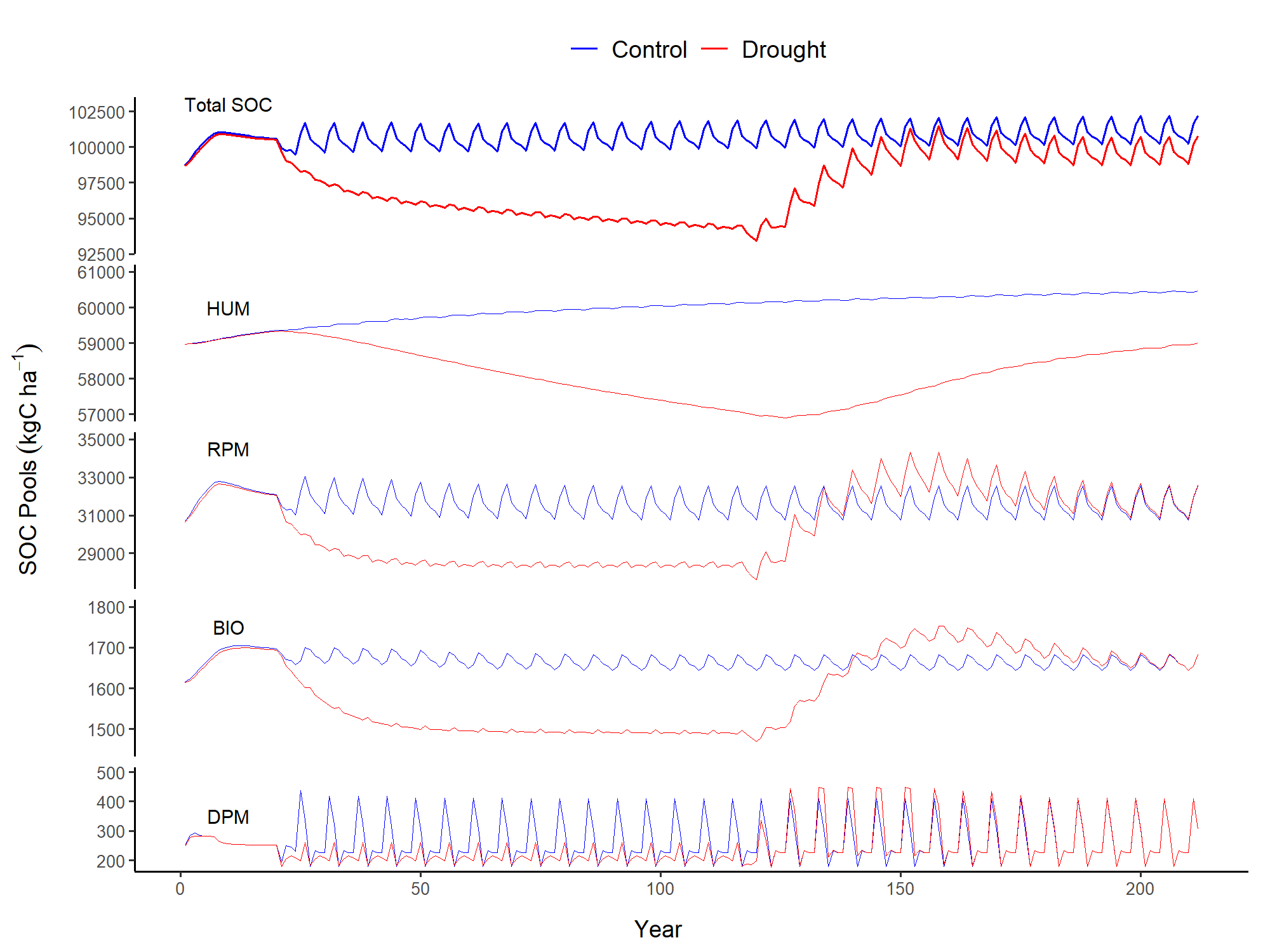


Figure S7: Simulation in the modelling scenario ‘96-year’ in the control and drought treatments of total soil organic carbon (Total SOC) and its corresponding active pools (decomposable plant material (DPM), resistant plant material (RPM), soil biomass (BIO), humic organic matter (HUM).


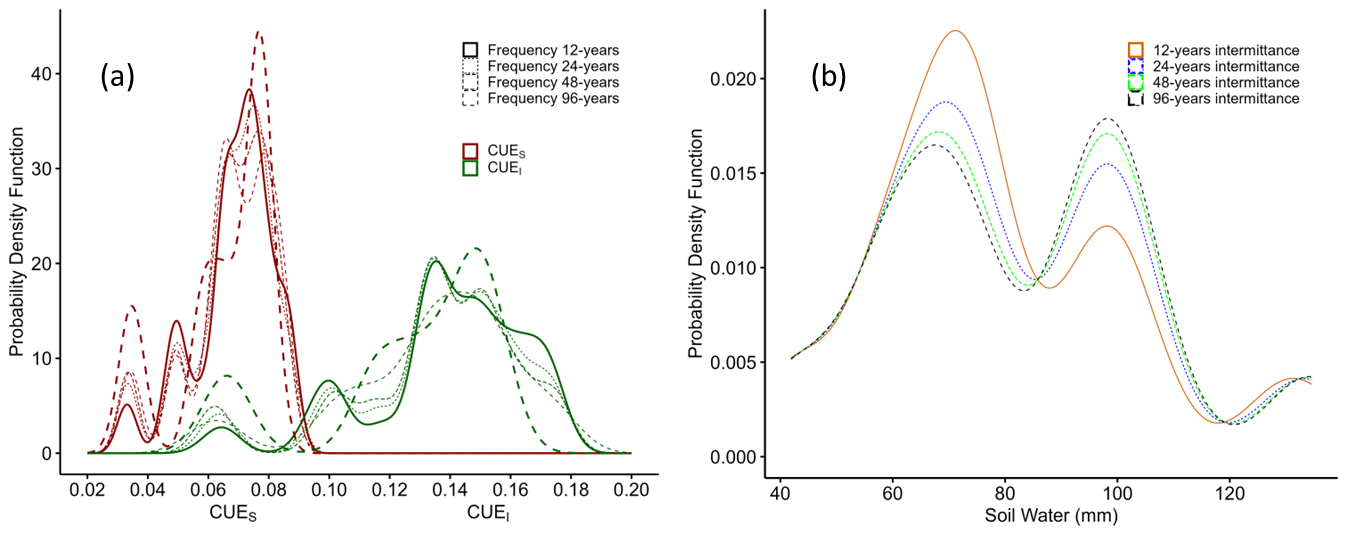


Figure S8: (a) shows the frequency distribution of carbon use efficiency (CUE) related to the decomposition of labile SOC pools (CUE_I_) and recalcitrant SOC pools (CUE_S_), and (b) shows the frequency distribution of soil available water for drought scenarios of different lengths.

**References**

Bai, Y., Wagener, T., Reed, P. (2009) A top-down framework for watershed model evaluation and selection under uncertainty. Environmental Modelling & Software 24, 901-916.

Bradbury, N.J., Whitmore, A.P., Hart, P.B.S., Jenkinson, D.S. (1993) Modeling the Fate of Nitrogen in Crop and Soil in the Years Following Application of N-15-Labeled Fertilizer to Winter-Wheat. Journal of Agricultural Science 121, 363-379.

Hopkins, D.W., Shiel, R.S., O'Donnell, A.G. (1988) The influence of sward species composition on the rate of organic matter decomposition in grassland soil. Journal of Soil Science 39, 385-392.

Lieth, H., (1975) Modeling the Primary Productivity of the World, in: Lieth, H., Whittaker, R.H. (Eds.), Primary Productivity of the Biosphere. Springer Berlin Heidelberg, Berlin, Heidelberg, pp. 237-263.

Manzoni, S., Porporato, A. (2009) Soil carbon and nitrogen mineralization: Theory and models across scales. Soil Biology & Biochemistry 41, 1355-1379.

Sierra, C.A., Müller, M. (2015) A general mathematical framework for representing soil organic matter dynamics. Ecological Monographs 85, 505-524.

Smith, P., Smith, J.U., Powlson, D.S., McGill, W.B., Arah, J.R.M., Chertov, O.G., Coleman, K., Franko, U., Frolking, S., Jenkinson, D.S., Jensen, L.S., Kelly, R.H., Klein-Gunnewiek, H., Komarov, A.S., Li, C., Molina, J.A.E., Mueller, T., Parton, W.J., Thornley, J.H.M., Whitmore, A.P. (1997) A comparison of the performance of nine soil organic matter models using datasets from seven long-term experiments. Geoderma 81, 153-225.

Thornthwaite, C.W. (1948) An Approach Toward a Rational Classification of Climate. Soil Science 66, 55-94.
